# Identifying key risk factors for intentional self‐harm, including suicide, among a cohort of people prescribed opioid agonist treatment: A predictive modelling study

**DOI:** 10.1111/add.70095

**Published:** 2025-05-25

**Authors:** Nicola R. Jones, Matthew Hickman, Chrianna Bharat, Suzanne Nielsen, Sarah Larney, Nimnaz Fathima Ghouse, Julia Lappin, Louisa Degenhardt

**Affiliations:** ^1^ National Drug and Alcohol Research Centre, University of New South Wales Sydney Australia; ^2^ SydneyMSK Research Flagship Centre, University of Sydney Sydney Australia; ^3^ Population Health Sciences Bristol Medical School, University of Bristol Bristol UK; ^4^ Monash Addiction Research Centre, Eastern Health Clinical School, Monash University Melbourne Australia; ^5^ Department of Family Medicine and Emergency Medicine Université de Montréal Montréal Canada; ^6^ Centre de Recherche du Centre Hospitalier de l'Université de Montréal Montréal Canada

**Keywords:** data linkage, machine learning, opioid‐related disorders, self‐harm, suicide & feature analysis

## Abstract

**Background and aims:**

People with opioid use disorder are at increased risk of intentional self‐harm and suicide. Although risk factors are well known, most tools for identifying individuals at highest risk of these behaviours have limited clinical value. We aimed to develop and internally validate models to predict intentional self‐harm and suicide risk among people who have been in opioid agonist treatment (OAT).

**Design:**

Retrospective observational cohort study using linked administrative data.

**Setting:**

New South Wales, Australia.

**Participants:**

46 330 people prescribed OAT between January 2005 and November 2017.

**Measurements:**

Intentional self‐harm and suicide prediction within a 30‐day window using linked population datasets for OAT, hospitalisation, mental health care, incarceration and mortality. Machine learning algorithms, including neural networks and gradient boosting, assessed over 80 factors during the last 3, 6 and 12 months. Feature visualisation using SHapley Additive exPlanations.

**Findings:**

Gradient boosting identified 30 important factors in predicting self‐harm and/or suicide. These included the most recent frequency of emergency department presentations; hospital admissions involving mental disorders such as borderline personality, substance dependence, psychosis and depression/anxiety; and recent release from incarceration. The best fitting model had a Gini coefficient of 0.65 [area under the curve (AUC) = 0.82] and was applied to 2017 data to estimate the probability of self‐harm and/or suicide. On average 46 people (0.16%) (from a total of 28 000 people in OAT) experienced intentional self‐harm or suicide per month. Applying a 0.15% probability threshold, approximately 5167 people were classified as high risk, identifying 69% of all self‐harm or suicide cases per month. This figure reduced to 450 per month after excluding people already identified in the previous month.

**Conclusions:**

Among people in opioid agonist treatment, administrative linked data can be used with advanced machine learning algorithms to predict self‐harm and/or suicide in a 30‐day prediction window.

## INTRODUCTION

Suicide and intentional self‐harm are complex public health challenges. Globally, it was estimated in 2019 that there were more than 700 000 suicide deaths each year [1], and for every person who dies by suicide, it is estimated that 20 times as many deliberately self‐harm [2]. People with opioid dependence are at elevated risk of suicide and self‐harm compared with the general population [3, 4, 5, 6]. Definitions of non‐fatal self‐harm are varied, but can include non‐suicidal self‐injury and non‐fatal suicide attempts. In clinical settings, it can be difficult to differentiate between self‐harm and suicidal intents [7, 8]. An emerging research area for suicide prevention, which is yet to be applied to people with opioid dependence, is to identify periods of service contact when these events are more likely to occur [9, 10, 11, 12].

Opioid agonist treatment (OAT) with methadone or buprenorphine is a World Health Organization essential medicine for the treatment of opioid dependence/use disorder, with wide‐ranging benefits including reduced all‐cause and overdose mortality in prison and community [13, 14, 15, 16, 17]. Increasing evidence also supports OAT effectiveness in reducing suicide and self‐harm, although as with opioid overdose the evidence also suggests that there may be elevated risk at specific periods of OAT induction and exit [9, 10]. The majority of intentional self‐harm and suicide events do not involve opioids [9].

It is hypothesised that identifying OAT clients most at risk will facilitate better targeting of self‐harm and suicide prevention and monitoring services [18]. Although risk factors for suicide and self‐harm are well known, most tools for identifying individuals at highest risk of these behaviours have limited clinical value because of low sensitivity, potentially from having a limited set of predictors [19, 20, 21]. For example, Huang *et al*. [22] concluded that demographic factors alone, although statistically significant, were not clinically significant [23]. Intentional self‐harm is a known predictor of suicide that should trigger additional assessment and intervention, but alone is insufficient to identify the rest of the population at risk of suicide. A recent review identified three critical considerations for progressing this area of research, two of which we implement in the present study [21]. First, timing, which to be clinically useful, algorithms should identify people at immediate risk who are already or recently engaged with services. Second, clinical utility requires the comparison and evaluation of the net benefit [24] of an algorithm. The third recommendation concerns piloting and implementation following the identification of a screening model.

Conventional methods have looked at risk factors as main effects, here, we explore the value of an alternative approach that can consider many potential predictors without the restrictions of inferential statistical theory. The term machine learning was introduced in the 1950s and, because of the evolution of computer processors, has now become commonplace. Machine learning techniques provide scalable solutions and are being applied to big health care data [25]. There are several kinds, supervised learning, used in this study, requires a historic dataset of predictors and outcomes to classify new cases where the outcome is unknown, a predictive model.

Here, we apply these techniques to determine if administrative data, which will include variations in clinical coding, can predict the combined short‐term self‐harm and suicide risk among people who have previously been in OAT for opioid dependence. We aim to determine an appropriate probability threshold to begin screening of people who are most at risk of self‐harm and/or suicide.

## METHOD

This was a retrospective cohort study using state‐wide linked administrative health data from New South Wales (NSW), Australia. Approval for this study was obtained from the NSW Population and Health Services Research Ethics Committee, and the Aboriginal Health and Medical Research Council Ethics Committee. Full details of the study, setting, data sources and linkage approach have been reported previously [26, 27]. Formulas and explanations are provided in Table S1, Supplementary S1.

The model building process followed closely to the cross‐industry standard process for data mining (CRISP DM) [28] methodology, which breaks down the task into six phases: the business understanding, data understanding, data preparation, modelling, evaluation and finally model deployment. A final step would be to continue to assess the effectiveness of a deployed model because of model degradation with environmental changes. We have used data between 2005 and 2017 to train, validate and test multiple machine learning algorithms, detailed below in the Machine learning; Model build, assessment and feature evaluation sub‐section. The best fitting algorithm was used to use calculate predictions for each month in 2017 to quantify the net benefit expected based on a range of probability thresholds, see Machine learning; Net benefit. Figure S1, Supplementary S2, illustrates the methodology. One limitation of machine learning algorithms is if left to train for too long on a dataset they will fit too well. This is known as overfitting and the model will not generalise well to new data. There are techniques available to prevent overfitting during model training by selecting models using a dataset not used to build the models [29].

### Setting and data sources

NSW, Australia, provides care for over one‐third of all people receiving OAT in Australia [30]. Methadone and buprenorphine are prescribed and dispensed in multiple settings for the treatment of opioid dependence, including public and private outpatient clinics and primary care, pharmacies and hospitals, as well as in prisons.

The NSW Electronic Recording and Reporting of Controlled Drugs (ERRCD) system is used in the administration of the NSW Opioid Treatment Program. For this study we used all available linked state‐wide:

- Admitted Patient Data Collection (APDC): admitted patient services provided by public hospitals (including psychiatric hospitals), private hospitals and private day procedures centres.
- Emergency Department Data Collection (EDDC): patient presentations to emergency departments (ED) of public hospitals [31].
- Mental Health Ambulatory Data Collection (MHAmb): mental health care contacts with non‐admitted patients, including mental health day programs, outreach services, community health service contacts and outpatient psychiatric contacts.
- Re‐offending Database, including custody data (ROD): finalised legal actions and adult and juvenile custody data.
- Death data: National Death Index (NDI) is administered by the Australian Institute of Health and Welfare and records deaths in Australia since 1980.

### Cohort

We have included all people in NSW who between January 2005 and November 2017 had at least 1 day of OAT. Observation commenced either on the 1 January 2005 (for people who commenced OAT before 2005) or the date exactly 12 months before each client's first recorded OAT treatment episode (for people commencing from 2005 onward). Observation ended on 30 November 2017, the date of death or 4 years post the cessation of the final treatment episode, whichever was earlier.

### Prediction window

A prediction window of 30 days was selected to build a model that captures imminent risk. All possible 30‐day prediction windows (the period at which self‐harm and\or suicide were predicted to occur) were generated and randomly sampled per participant, from any time after the initial OAT treatment commencement or January 2005 (in the case of people who started OAT before this date). Follow‐up ended after 30 days, on 30 November 2017 or if death occurred. We chose 3, 6 and 12 months look back periods. Anything beyond 12 months was considered not recent enough, and the 6 months served as a midpoint. We did not include a more recent look‐back period to allow for a possible delay in data availability in a real‐world setting.

### Outcome of interest

The primary outcome during the prediction window was: (1) suicide deaths derived from the NDI; and (2) having one or more intentional self‐harm hospitalisations (include non‐suicidal self‐injury) derived from APDC and EDDC records.

Adapted from previous studies [9, 10, 32], coding from the International Classification of Diseases 10th edition (ICD‐10‐AM) was used to identify suicide and intentional self‐harm using the hospital data and ED presentations. The recording of suicidal intent can be difficult to determine. We chose to exclude all self‐harm of undetermined intent to ensure model precision. Primary and additional diagnoses and/or contributory fields were interrogated.

ED presentations were clinically coded using ICD‐10‐AM, ICD‐9 or Systemized Nomenclature of Medicine Clinical Terms (SNOMED‐CT). All codes used to derive the outcome are given in Table S2.

### Features

Model features were identified and derived from each data collection based on their conceptual association with intentional self‐harm and established statistical significance in other studies [9, 10, 32].

#### Socio‐demographics

These include sex (1 = female and 2 = male), Aboriginal and/or Torres Strait Islander status (1 = Indigenous, 0 = not Indigenous), age at time of prediction, geographical remoteness and socio‐economic disadvantage index. Residential remoteness (major cities vs. regional/remote) was based on each participants' last known postcode of residence using the Accessibility/Remoteness Index of Australia Plus (ARIA+) 2016 (Australian Bureau of Statistics), along with socio‐economic disadvantage that was based on SEIFA (Australian Bureau of Statistics). An indicator for homelessness was derived from hospital records for the previous 3‐, 6‐ and 12‐months.

#### Self‐harm co‐morbidities

Previous hospitalisations for overdose and poisoning by intent and data source (APDC or EDDC), along with incidence of chronic pain and self‐inflicted injury were derived in the last 3‐, 6‐ and 12‐months. Intentional, accidental and unknown intent events were separated.

#### Mental health co‐morbidities

ED, inpatient and outpatient hospital data were used to define conditions of dependence by substance, depression and anxiety, suicide ideation, borderline personality disorder, psychosis and PTSD, and were derived for the previous 3‐, 6‐, and 12‐months.

#### Treatment

Time since index OAT, number of days on OAT, number of prescribers (as an indication of medication stability) and number of OAT episodes in the last 3‐, 6‐ and 12‐months were derived.

#### Criminographic history

Release from incarceration over the last 3‐, 6‐ and 12‐months was derived by type of charge (violent vs. non‐violent). A full list of definitions is provided in Tables S3–S5.

### Machine learning

Several complex techniques have been used in this study. The model building process followed closely to the CRISP DM [28] methodology, which breaks down the task into six phases as detailed above.

#### Sampling

Because of the rare nature of our outcome of interest (<1% of the population) a technique called separate sampling (oversampling) was applied. Separate sampling involves retaining all records of events of self‐harm/suicide, and a random selection from the non‐event records. For each person experiencing an event, one prediction window was randomly selected for the calendar month of the event date. A random selection of non‐event records per person per year was selected and the two datasets were combined to create an oversampled data table.

The features detailed in the section above were derived from the day before the generated start date of the 30‐day prediction window. The data table was randomly partitioned 40:30:30 into three smaller tables, stratified by outcome: [TRAINING] for building the models; [VALIDATION] for tuning model parameters; and [TEST] a hold‐out sample for testing a model's generalisability to data not used during model development. Greater detail is provided in Supplementary S1, Sampling and Adjustment for separate sampling sections.

#### Model build and assessment

Four classes of model were fitted to the features selected: logistic regression; multilayer perceptron (MLP) neural network; gradient boosting (GB); and ensemble. During the development phase these models are considered candidate models. The model selected for deployment is termed the champion model. See Supplementary S1, Model build and assessment section.

Propensity scores, across TRAINING, VALIDATION and TEST datasets, were generated from each of the algorithms. Model performance was assessed in terms of discrimination and calibration in the holdout sample [TEST], the Gini coefficient was used as the primary measure of goodness of fit. The Gini coefficient is an index for the degree of inequality in the distribution of the positive prediction rate, it ranges from 0 to 1 and is used to estimate how far a model is capable of deviating from a uniform distribution (i.e. selecting from random). The area under the receiver operating characteristic (ROC) curve (AUC), which is related to the Gini coefficient was also reported as it is more commonly used. A champion model was chosen based on the largest Gini coefficient.

#### Feature evaluation

The important features for the best model were investigated. Variable importance can be measured when using the GB algorithm, a split‐based approach summing the reduction in the sum of squares from splitting a node, over all nodes. A node is where a data partitioning rule is applied. For models, such as neural networks, ‘black boxes’, two additional methods of model interpretation were used. The first, fitting a decision tree to the predictions generated from the algorithm, the predictions would need to be transformed into a binary variable by applying a threshold probability to determine highest risk. The second, is a recent technique, Shapley additive explanations (SHAP) developed by Scott M. Lundberg [33]. SHAP uses Shapley values derived from game theory [34], and measures the impact of variables considering the interaction with other variables.

#### Net benefit

The measurement net benefit [24] was used to compare the overall advantage gained from deploying a statistical model compared to not using it in terms of improved decision‐making. Net benefit involves weighing elements such as precision, cost benefit, risk assessment and scalability. An exchange rate is used to transform the benefits and harms at each threshold probability to a simple assessment measure for model comparison. The exchange rate is a clinical judgement of the relevant value of detection and harms (misclassification), it is where the expected benefit of treatment is equal to the expected benefit of avoiding treatment. For clinical prediction models, the exchange rate is mapped to the threshold probability to decide whether we expect an event in the next 30 days.

We compared the benefit of undertaking self‐harm/suicide screening using the intervention for all people at risk approach (the threshold is a proportion of a random selection of the cohort) with; (1) the prediction model; and (2) using a rule‐based measure of the two most important markers, (1) more than one emergency presentation in the last 6 months; (2) a hospitalisation for intentional overdose in the last 12 months; and (3) using both markers as an inclusive disjunction. More details are provided in Supplementary S1, Net benefit.

For this comparison, we used all cohort records in 2017, using the first 30 days of each calendar month to provide estimates of how this could work in a real‐world implementation for NSW. The champion model was applied to all people in the OAT cohort for each month in 2017 to calculate the probability of intentional self‐harm/suicide in the 30‐day window. A threshold probability for classifying people at highest risk is required as well as the feasibility of the number of people to screen to find at least one person at risk of self‐harm/suicide. The overall advantage of a decision to intervene was quantified by calculating total benefits minus total costs.

Decision curves plotting net benefit against multiple threshold probabilities were created. The purpose of the decision curve is twofold: (1) to measure each strategy, the strategy with the highest net benefit at a particular threshold probability has the highest clinical value; and (2) to determine which is the optimal threshold to implement.

Data set construction was done using SAS version 9.4 (SAS Institute). SAS Enterprise Miner V14.3 was used for model comparisons. R Statistical Software (v4.1.2; R Core Team 2021) was used for feature visualisation, data was imported using the haven package [35] and visualisations used the fastshap package (v0.1.1; [36]).

The analysis was exploratory and not pre‐registered but any future testing of its implementation or evaluation of the probability threshold will be pre‐registered.

## RESULTS

There were 46 330 people in the cohort, cohort characteristics are provided in Table S9. On average there were 46 people experiencing at least one self‐harm/suicide event per month from approximately 28 539 participants with a history of OAT in the last 4 years (0.161% of the population in OAT) (Table 1).

**TABLE 1:** Cohort size.

Cohort year	Average monthly cohort size	Monthly average self‐harm events	Monthly average suicides	Monthly average all events	Monthly average cohort proportion with at least one outcome (%)
2005	28 151.3	40.1	2.1	42.2	0.150
2006	28 553.8	46.4	1.8	48.3	0.169
2007	28 496.4	44.8	2.3	47.0	0.165
2008	28 229.8	37.3	1.8	39.0	0.138
2009	28 287.9	41.8	1.9	43.8	0.155
2010	28 568.6	36.7	1.6	38.3	0.134
2011	28 729.8	39.3	2.0	41.3	0.144
2012	28 705.0	41.5	3.4	44.9	0.156
2013	28 890.6	46.8	2.3	49.0	0.170
2014	28 914.0	45.4	2.6	48.0	0.166
2015	28 649.5	47.1	3.6	50.7	0.177
2016	28 440.2	54.3	2.2	56.5	0.199
2017	28 385.8	45.6	2.4	48.0	0.169
Overall monthly average	28 538.7	43.6	2.3	45.9	0.161

There were 391 579 rows in the initial oversampled data table, of which 7268 (2%) were event records. Of the 7268 events, 6950 were self‐harm events and 366 suicides during the follow‐up period. A total of 3467 (0.89%) records were removed from the model building phase because of missing data. There were four records removed because of unknown sex, and a further 3463 for missing remoteness or socio‐economic disadvantage index because of missing/non‐matching postcode. This removed 48 event records.

### Model build and assessment

Goodness of fit statistics for the three partitioned datasets from fitting the machine learning algorithms are provided in Table S8 and Figure S2. The GB model returned the highest Gini coefficient (0.65) equivalent to ROC of 0.82. The GB algorithm built 600 subtrees before convergence was met and no further improvement was found. Each step was evaluated using the validation data, a 320‐subtree model had the lowest assessment statistic for the VALIDATION, this model was chosen.

### Feature evaluation

There were 30 features used in the selection process. The most important feature was whether there had been a hospitalisation for intentional overdose in the last 12 months, followed by the number of ED presentations in the last 6 months, and the third most important was a hospitalisation for a poisoning of unknown intent in the last 12 months. ED presentations in the past 6 months were the most important interaction feature (Table S10).

A threshold probability percentage of 1.86% (sample event rate) was used to classify highest risk from the probabilities generated using the training dataset from the GB algorithm. This resulted in 16.8% of the cohort being identified as at highest risk. Using a sample of 10 000, a decision tree was built that captured groups with high percentages of our outcome and are illustrated in Figure S3. The number of ED presentations in the previous 6 months was frequently used throughout the partitioning. There was a fourfold increase, (an 8.9% event rate), for people with more than one ED in 6 months, this rule alone captured 46.7% of events. The rule with the purest partitioning, with a fivefold increase in the percentage of people with the event, a 9.7% event rate, were people with: (1) no ED presentations in the previous 6 months, but with (2) hospitalisation for both unknown intent poisoning and intentional overdose in the last 12 months; and (3) no outpatient for opioid dependence in the previous 12 months or hospitalisation for other substance dependence or depression and\or anxiety. However, this rule only captures less than 1% of all events.

The results from the GB variable importance and SHAP were comparable. The number of ED presentations in the last 6 months had the highest average SHAP score (0.531) the values were spread wide (high feature values had a high SHAP).

**FIGURE 1:**
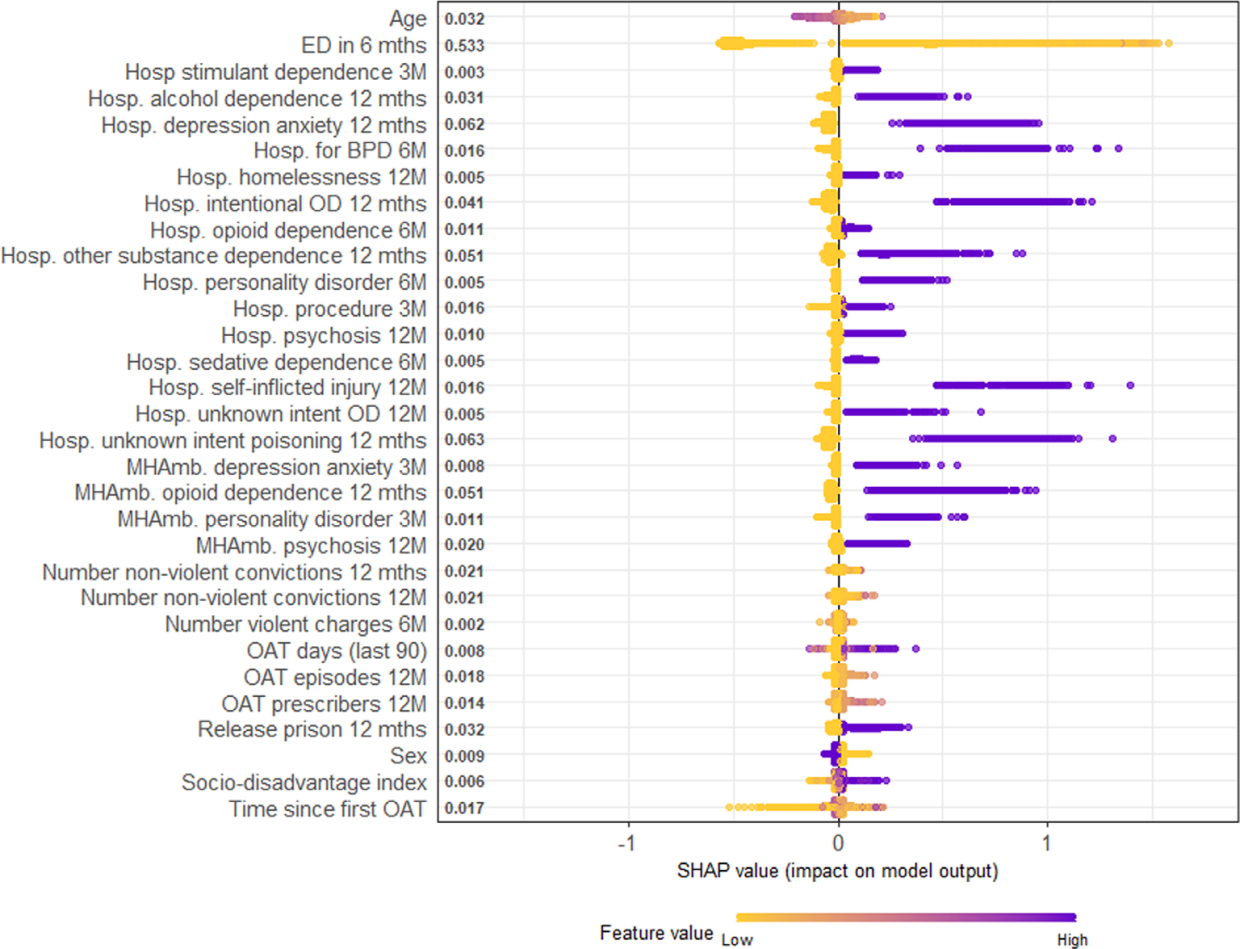
Shapley additive explanations (SHAP) feature impact. The figure is based on a sample of *n* = 25 000, each point is a Shapley value for a feature and an observation. The position on the y‐axis is determined by the feature and on the x‐axis by the Shapley value. The colour represents the value of the feature, here, many of the features are Boolean, and therefore, a value of 1 is purple in colour.

There are clear divisions regarding feature values and the impact on the model. People experiencing either a hospitalisation for; self‐inflicted injury, intentional overdose, depression and\or anxiety or an unknown poisoning in the last 12 months; borderline personality disorder in the last 6 months; or a mental health outpatient visit for personality disorder in the last 6 months, all these factors contribute to the prediction score positively (Figure 1). A release from incarceration or being female positively contributes to the probability score.

**FIGURE 2:**
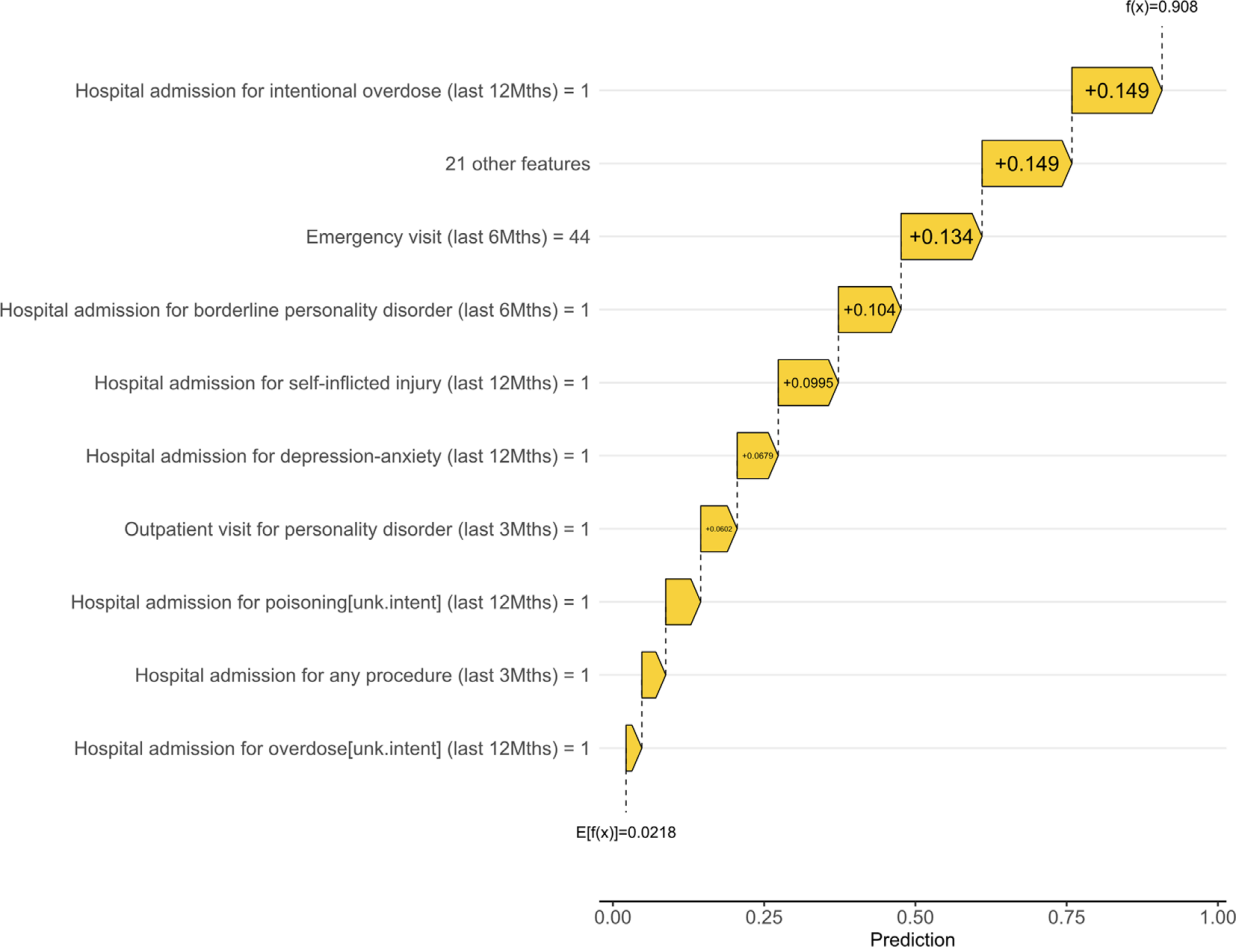
Individual A—Shapley additive explanations (SHAP) waterfall. The horizontal axis measures the amount each feature contributes to the overall probability to intentional self‐harm/suicide. Each feature has an arrow, the direction represents whether it positively (right) or negatively (left) contributes to the probability. The length of the arrow represents the magnitude of the impact. E[f(x)] represents the average of all predictions and f(x) represents the prediction for individual A.

A waterfall visualisation of the Shapely scores for a high‐risk participant from the TEST dataset is shown in Figure 2 and emphasises the diversity of the features. The expected probability of self‐harm/suicide on average was *P* = 0.0216. The model correctly predicted individual A as highest risk, probability of 0.911 (adjusted probability of 0.481). The three main factors contributing to the probability score were: a hospitalisation for intentional overdose in the previous 12 months; the number of ED in the previous 6 months (*n* = 44); and a hospitalisation for borderline personality disorder in the previous 6 months. Further examples are given in Figures S4–S6.

### Net benefit

**FIGURE 3:**
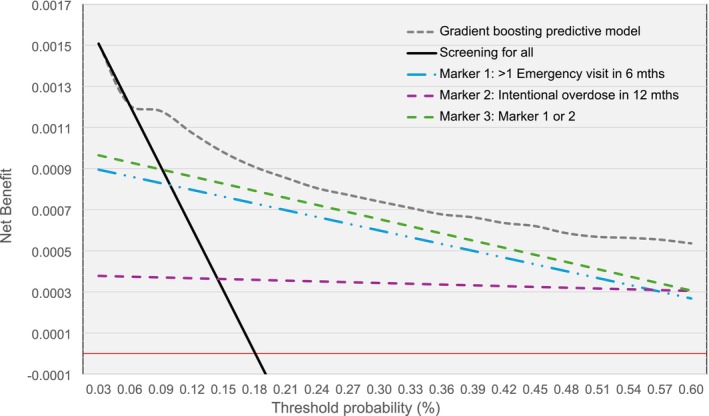
Net benefit decision curve. See Table S11 for gradient boosting sensitivity and specificity.

The net benefit, a measure of both harm and benefit, was used to determine whether making clinical decisions using a model on who to screen could do more good than harm, compared to screening for all at multiple threshold probabilities. The decision curves of the net benefit for each strategy across threshold probabilities, ranging from 0% to 0.6% are provided in Figure 3, (the net benefit drew close to 0 at 10%).

The strategies of screening for all and prediction model were comparable up to a probability threshold of 0.06%. The marker models had lower net benefit than the screening for all strategy for threshold probabilities 0% to 0.1%, and the best clinical outcome would be achieved by randomly selecting from all observations irrespective of markers. From a threshold probability of 0.06% the net benefit for the GB model demonstrates higher net benefit than the treatment for all approach and was better than the marker strategies 1 to 3.

As the threshold probability increases, the number of people identified decreased along with the percentage of people correctly identified as high risk. At a threshold probability of 0.15%, 69% (Table S11) of people who self‐harmed/suicide in the 30‐day window were correctly identified by the model, from a subset of 18% (*n* = 5160) of the monthly cohort. At this threshold sensitivity was 0.69, with a specificity of 0.84. Throughout 2017 there were 4646 unique cohort members identified that changed to a high‐risk classification, an average of 450 per month compared to 5160 per month people in total, 1.18% self‐harmed within 3 months (7 times higher than baseline). The predictive model provided the greatest net benefit, and the probability scores can be used to identify those at the greatest risk and by selecting different thresholds the implementation can be scaled to align with available resources.

## DISCUSSION

We have shown that administrative linked data can be used with advanced machine learning algorithms to predict self‐harm and/or suicide in a 30‐day prediction window. The best‐fitting model was a GB algorithm that included ED presentations in the last 6 months, history of intentional overdose in the last 12 months and multiple touchpoints with hospitals (inpatient and outpatient) for personality disorders among several other predictors. The model AUC was above 0.8, with sensitivity of 0.69 and specificity of 0.84, and Gini coefficient at 0.65 could be considered as ‘very good’ in accordance with previous models. [37] A strength of GB is their ability to identify non‐linear relationships and learn from its errors (residuals), therefore, it can identify intricate patterns and is not dependent on strong predictors such as a history of self‐harm, such as the individual identified as high risk in the Supplementary S6.

With a probability threshold percentage set at 0.15% the model identified on average 5160 of a total 28 000 people in NSW per month for targeted screening and/or self‐harm prevention interventions, approximately 450 people per month who were not flagged the previous month. It is superior in terms of discriminatory power to simpler algorithms and likely to be more cost‐effective than screening all patients (a monthly average of 28 000). The theory of suicide being an aleatory event is likely the cause of the low‐sensitivity in predictive models, to identify more cases the net has to go wider and some events cannot be predicted [38, 39].

### Feature complexity

Previous studies have identified a history of self‐harm and mental health problems as major risk factors for overdose and suicide among people who use opioids, consistent with this study. A review of suicide prediction models in 2019 concluded that larger predictor datasets were required to predict suicide effectively, to be used in conjunction with staged screening designs (such as self‐report surveys), the use of machine learning and evaluation using net benefit [21]. Because of the large cohort size, we were able to assess over 230 predictors from 82 main factors to identify people at highest risk. This has provided a key advantage over small studies with insufficient power to determine the importance of the many contributing factors examined.

Although it is generally agreed that self‐harm is associated with higher suicide risk, and therefore, should trigger an intervention, people who self‐harm/suicide are a heterogeneous population making risk identification a complex task. This study has identified additional factors such as the number of ED presentations and the number of prescribers of OAT in the previous 3 months that may increase our ability to identify those at risk.

This study was a prototype, a pilot study designed for production data and a method of model deployment is a feasible next step.

### Implementation

The model is the first step in assessing the predictive ability of using administrative data to identify people at risk. Most of the important features identified in this model were from hospital records and are already accessible in an integrated health record within the NSW healthcare system, alongside other healthcare systems working toward a single digital patient record. Classifying people at risk is architecturally a simple solution and has the advantage of identifying people in a setting in which they could receive additional support/clinical care that may prevent likely future harm and healthcare admissions. In many existing healthcare systems using Electronic medical records, this would be a feasible trigger to flag in the system. People who meet the risk criterion on presentation at hospital would be automatically flagged and relevant risks could then be assessed with appropriate clinical care offered. It is very likely that an identical data structure will not be available in all drug and alcohol clinics. However, linkage between drug and alcohol and other hospital services is growing. Therefore, an analytical data table will need to be built as close to real time as possible and models will need to be rebuilt and tested if predictors identified here are not available.

The Shapley scores enable a clinician to understand which features have contributed to an individual's high‐risk assessment (positive as well as negative), which may be beneficial.

### Strengths and limitations

Advances in computational power and electronic data capture have provided opportunities in healthcare to apply novel solutions and transform the current landscape. The use of machine learning is on the increase, but its lack of interpretability and complexity impedes its adoption in a clinical setting. Machine learning algorithms are not bound by assumptions, they can predict very complex relationships and rely on the CRISP‐DM [28] methodology to assess and prevent overfitting to ensure that the model generalises well to a new set of observations.

The key limitation is the availability of the data—which in this study required the merging of seven data sets in real time to deploy the algorithm. Information on interventions to prevent self‐harm were not available within the dataset. Administrative data are not collected for the purpose of predicting self‐harm and/or suicide. Reliability of some of the factors may be compromised. Ideally features using health diagnosis codes could be refined further to improve accuracy. For example, chronic pain was derived using 151 SNOMED‐CT codes (a recent study has shown a far more comprehensive method for defining chronic pain) [40]. In addition, there are several important features that were unavailable such as ambulance callouts, employment status or highest education qualification, which have been identified as important factors in predicting self‐harm/suicide [41].

## CONCLUSIONS

It is not possible to screen all people in OAT for risk of self‐harm. Instead, we show that it could be feasible to use machine learning with clinical and administrative data to identify a smaller set of people in OAT with higher risk for intentional self‐harm and suicide for further screening and targeting of additional interventions for prevention.

## AUTHOR CONTRIBUTIONS

**Nicola Jones**: Conceptualization (lead); data curation (equal); methodology (equal); formal analysis (lead); visualization (equal); writing—original draft (lead); writing—review and editing (lead). **Matthew Hickman**: Conceptualization (equal); methodology (equal); writing—original draft (equal); writing—review and editing (equal). **Sarah Larney**: Conceptualization (equal); methodology (equal); writing—original draft (equal); writing—review and editing (equal). **Chrianna Bharat**: Conceptualization (equal); methodology (equal); formal analysis (supporting); visualization (equal); writing—original draft (supporting); writing—review and editing (supporting). **Suzanne Nielsen**: Conceptualization (equal); methodology (equal); writing—original draft (equal); writing—review and editing (equal). **Julia Lappin**: Writing—review and editing (equal). **Nimnaz Fathima Ghouse**: Data curation (equal). **Louisa Degenhardt:** Conceptualization (equal); funding acquisition (equal); data curation (equal); resources (lead); writing—original draft (supporting).

## DECLARATION OF INTERESTS

None.

## Supporting information


**Table S1.** Glossary.
**Table S2.** Self‐harm and suicide ICD‐10, 9 and SNOMED codes.
**Table S3.** Predictor definitions.
**Table S4.** ICD and SNOMED codes used for health service utilisation.
**Table S5.** Criminal charges derivation.
**Table S6.** Dimension reduction – results from decision tree.
**Table S7.** Machine learning algorithm properties.
**Table S8.** Model goodness of fit statistics. The gradient boosting algorithm had the highest Gini coefficient based on the test (holdout) dataset, this model differentiates best between those that will self‐harm/suicide from those that will not.
**Table S9.** Cohort characteristics at first prediction window.
**Table S10.** Gradient boosting important features. Note: (*DATA source‐variable type‐time period*). The importance of a variable is the contribution it makes to the success of the model. A high ratio of validation to training importance for a variable suggests that the variable is important not only in the training data but also in the validation data, indicating good generalization.
**Table S11.** Model net benefit, sensitivity and specificity per threshold probability. At a threshold probability of 0.15, i.e. a probability of self‐harm/suicide > = 0.15, the model identified 5167 at high risk, with sensitivity of 69% and specificity of 84%. The net benefit had a value of 0.00098 for the predictive model compared to 0.00031 for the screen for all approach.
**Figure S1.** Prediction model process of self‐harm/suicide within any 30‐day window.
**Figure S2.** Sensitivity versus 1‐specificity (ROC) chart. Note: Sensitivity, [# of true positives/(# of true positives + # of false negatives)], is the ability of a model to correctly classify an individual as being hospitalised for self‐harm within a 30‐day window. Specificity the ability of a model to correctly classify an individual as NOT being hospitalised for self‐harm or death by suicide within a 30‐day window, [# of true negatives/(# of true negatives + # of false positives)].
**Figure S3.** Decision tree output, predicting the gradient boosting outcome using a sample (n = 10 000) .
**Figure S4.** Individual B ‐ SHAP waterfall. This individual had an expected probability to self‐harm/suicide of 0.681 much higher than the baseline risk of 0.0218. The top factors that contributed to this high risk were a hospitalisation with a diagnosis of borderline personality disorder in the last 6 months, a hospitalisation for an intentional overdose in the last 12 months as well as self‐inflicted injury.
**Figure S5.** Individual C ‐ SHAP waterfall. This individual had an expected probability to self‐harm/suicide of 0.541, the top factors that contributed to this high risk were 19 ED visits in the last 6 months, a hospitalisation for an intentional overdose in the last 12 months as well as poisoning (unknown intent).
**Figure S6.** Individual D ‐ SHAP waterfall. This individual had an expected probability to self‐harm/suicide of 0.0324, the top factors that contributed to a higher risk than baseline (0.0218) were an outpatient visit for opioid dependence, recent hospitalisation for substance dependence and psychosis. Having no ED visits in the last 6 months reduced their propensity score.

## Data Availability

Data subject to third party restrictions.
