# Supplementary material for: Identifying key risk factors for intentional self‐harm, including suicide, among a cohort of people prescribed opioid agonist treatment: A predictive modelling study
Source: Addiction. 2025 May 25;120(10):2044–54. doi: 10.1111/add.70095 (PMC12426353; doi:10.1111/add.70095)
Supplement: Supplementary file 1 — Table S1. Glossary. Table S2. Self‐harm and suicide ICD‐10, 9 and SNOMED codes. Table S3. Predictor definitions. Table S4. ICD and SNOMED codes used for health service utilisation. Table S5. Criminal charges derivation. Table S6. Dimension reduction – results from decision tree. Table S7. Machine learning algorithm properties. Table S8. Model goodness of fit statistics. The gradient boosting algorithm had the highest Gini coefficient based on the test (holdout) dataset, this model differentiates best between those that will self‐harm/suicide from those that will not. Table S9. Cohort characteristics at first prediction window. Table S10. Gradient boosting important features. Note: (DATA source‐variable type‐time period). The importance of a variable is the contribution it makes to the success of the model. A high ratio of validation to training importance for a variable suggests that the variable is important not only in the training data but also in the validation data, indicating good generalization. Table S11. Model net benefit, sensitivity and specificity per threshold probability. At a threshold probability of 0.15, i.e. a probability of self‐harm/suicide > = 0.15, the model identified 5167 at high risk, with sensitivity of 69% and specificity of 84%. The net benefit had a value of 0.00098 for the predictive model compared to 0.00031 for the screen for all approach. Figure S1. Prediction model process of self‐harm/suicide within any 30‐day window. Figure S2. Sensitivity versus 1‐specificity (ROC) chart. Note: Sensitivity, [# of true positives/(# of true positives + # of false negatives)], is the ability of a model to correctly classify an individual as being hospitalised for self‐harm within a 30‐day window. Specificity the ability of a model to correctly classify an individual as NOT being hospitalised for self‐harm or death by suicide within a 30‐day window, [# of true negatives/(# of true negatives + # of false positives)]. Figure S3. Decision tree output, p [file ADD-120-2044-s001.docx]

# Identifying key risk factors for self-harm and suicide among a cohort of people prescribed opioid agonist treatment: a predictive modelling study - Supplementary 1 & 2

Contents

[***Identifying key risk factors for self-harm and suicide among a cohort of people prescribed opioid agonist treatment: a predictive modelling study - Supplementary 1 & 2*** 1](#_Toc195463210)

[Supplementary 1 – Methodology 2](#_Toc195463211)

[Sampling 2](#_Toc195463212)

[Adjustment for separate sampling 3](#_Toc195463213)

[Dimension reduction 3](#_Toc195463214)

[Model build and assessment 3](#_Toc195463215)

[Net benefit 5](#_Toc195463216)

[Table S1 Glossary 7](#_Toc195463217)

[Table S2 Self-harm and suicide ICD-10, 9 and SNOMED codes 9](#_Toc195463218)

[Table S3 Predictor definitions 10](#_Toc195463219)

[Table S4 ICD and SNOMED codes used for health service utilisation 16](#_Toc195463220)

[Table S5 Criminal charges derivation 28](#_Toc195463221)

[Table S6: Dimension reduction – results from decision tree 30](#_Toc195463222)

[Table S7: Machine learning algorithm properties 33](#_Toc195463223)

[Supplementary 2 - Results 34](#_Toc195463224)

[Table S8: Model goodness of fit statistics 34](#_Toc195463225)

[Table S9: Cohort characteristics at first prediction window 34](#_Toc195463226)

[Table S10: Gradient boosting important features 35](#_Toc195463227)

[Table S11 Model net benefit, sensitivity and specificity per threshold probability 36](#_Toc195463228)

[Figure S3 Decision tree output, predicting the gradient boosting outcome using a sample (n=10000) 38](#_Toc195463229)

[Figure S4 Individual B - SHAP waterfall 39](#_Toc195463230)

[Figure S5 Individual C - SHAP waterfall 40](#_Toc195463231)

[Figure S6 Individual D - SHAP waterfall 41](#_Toc195463232)

[References 41](#_Toc195463233)

## Supplementary 1 – Methodology

The model building process followed closely to the CRISP DM (1) methodology which breaks down the task into six phases: the business understanding, data understanding, data preparation, modelling, evaluation and finally model deployment. A final step would be to continue to assess the effectiveness of a deployed model due to model degradation with environmental changes.

### Sampling

Due to the rare nature of our outcome of interest (<1% of the population), all people could be classified as low risk and we would be correct 99% of the time, a technique called *separate sampling (oversampling)* was applied. Separate sampling involves retaining all records of self-harm/suicide, and a random selection from the non-event records. For each cohort participant, all possible 30-day exposure periods were simulated from the follow-up period, this resulted in approximately 30 or 31 records per person per month. The outcome (self-harm and/or suicide) was derived in the 30-days following the start date – this interval is referred to as the ‘prediction window’. The data was separated into two, self-harm and/or suicide events separated from no event. For each person experiencing an event one of the 30-day window records was randomly selected for the calendar month of the event.

There were 4,717 possible predictions windows, 134,063,414 prediction windows in total for all OAT participants, 133,842,302 of these windows had no event. 1 window per person per year was chosen at random of the non-events, resulting in 387,730 prediction windows. 7967 prediction windows had an event, one per person per calendar month was chosen at random, to give 7162 prediction windows with an event. People who have experienced intentional self-harm will have one non-event prediction window per year and one prediction window per month for each self-harm event. On average each person was included in 9 prediction windows, with an interquartile range of 5-13.

The final table included 1.8% records with a self-harm event and the remaining did not, creating an oversampled data table.

The features detailed in the section above were derived from the day prior to the generated start date of the 30-day prediction window. The data table was randomly partitioned into 3 smaller tables, stratified by outcome: [TRAINING] for building the models; [VALIDATION] for tuning model parameters; and [TEST] a hold-out sample for testing a model’s generalisability and performance when applied to data not used during the model development process. The data table was partitioned 40:30:30 into TRAINING : VALIDATION : TEST.

### Adjustment for separate sampling

Given that the data table used in the analysis was oversampled, model posterior probabilities, $\hat{p}$*_i_*^*^, were adjusted using prior probabilities, π*_i_*, that represent the proportion of the cohort experiencing the outcome (*i*=1) and non-outcome, (*i*=0). The following formula was used to adjust probabilities for the scored dataset, the scored dataset was based on the OAT cohort in 2017. (ρ_i_ represents the proportion in the training dataset experiencing the outcome *i*).

$$\hat{p}_{i}= \frac{\hat{p}_{i}^{*}\rho_{0} \pi_{1}}{\left( 1-\hat{p}_{i}^{*} \right) \rho_{1}\pi_{0}+ \hat{p}_{i}^{*} \rho_{0} \pi_{1}}$$

### Dimension reduction

Gradient boosting and logistic regression have built in method of variable selection but a neural network does not. To build an optimal neural network model, raw data transformations and the reduction of collinear and redundant variables are required. The training data will have many variables that are correlated especially due to the time element that has been built in, looking at the previous 3-, 6-, and 12-months.

Two methods of dimension reduction were considered to remove collinear and redundant predictors: 1) decision tree, a version of CHi-square Automatic Interaction Detection (CHAID), the tree stops partitioning when there are no more variables with an association to the outcome; and 2) variable clustering, this method groups correlated variables and passes the variable that explains the most variability within the group to the next stage.

In total 228 features were created, of these 11 were rejected due to being unary, i.e. all observations had the same value. The decision tree reduced the feature list from 228 to 125 features. Many features selected included a combination of the 3-, 6- or 12-month period, the most important were selected manually to be used in the following stage based on the results from the tree and the variable clustering dimension reduction technique. This reduced the number of features to 57, **Table S6**.

### Model build and assessment

Four classes of models were fitted to the features selected: Logistic regression; Multilayer perceptron (MLP) neural network; Gradient boosting; and Ensemble.

1. Logistic regression, although considered a traditional method, belongs to the family of supervised machine learning models. The regression model learns using a variable selection method such as stepwise model selection, at each step the model is evaluated using the validation dataset. The model trains using the training dataset testing each variable for entry into the model until it reaches the final set of predictors based on the model selection parameters provided, but typically a simpler model is chosen based on a goodness of fit statistic that is optimized based on the validation data.
2. The multilayer perceptron (MLP) neural network utilizes all available predictors as inputs to the model. It consists of multiple layers, known as hidden layers, which contain hidden units that perform non-linear transformations of the inputs. As the number of hidden layers increases, the number of weights in the model grows exponentially, contributing to the equation used to predict the desired output. While MLPs can effectively fit complex patterns in data, they also run the risk of overfitting.
3. Gradient boosting is a type of ensemble model, it is based on many decision trees that singulary are weak predictors but combined can be very effective. Instead of predicting the observed values the residuals are predicted. The aim is to sequentially add new models that correct the errors made by the previous ones. However, it can be sensitive to noise and prone to overfitting if not properly managed.
4. An ensemble model is a machine learning approach that combines multiple individual models to improve overall performance. The premise is that by combining the predictions of several models per observation, you can achieve better accuracy, robustness, and generalization than any single model could provide. Combination methods are average, maximum or voting. We have used maximum posterior probability across the models.

During the development phase these models are considered candidate models, the final model selected for implementation is termed a champion model. When models are deployed into a production environment sometimes a candidate model is also implemented and the performance of both models are assessed over time. Should the champion model degrade and the candidate model starts to perform better, the models can be switched over until a new set of models can be developed.

Propensity scores, across TRAINING, VALIDATION and TEST datasets, were generated for each of the algorithms. Model performance was assessed in terms of discrimination and calibration in the holdout sample [TEST], the Gini coefficient was used as the primary measure of goodness of fit. The Gini coefficient is an index for the degree of inequality in the distribution of the positive prediction rate, it ranges from 0 to 1 and is used to estimate how far a model is capable of deviating from a uniform distribution, i.e. selecting from random. A champion model was chosen based on the model with the highest Gini coefficient.

A more commonly known measure of goodness of fit was also estimated by means of generating the receiver operating characteristic (ROC) to aid in interpretation. The ROC visualises trade-offs in different aspects of performance as the threshold applied to a model’s prediction varies. The area under the ROC curve (AUC) provides a measure of discriminative ability and is related to the Gini coefficient.

The TRAINING dataset had 153,724 records, 2,907 (1.86%) were event records and the VALIDATION and TEST datasets had 115,293 and 115,294 records, 2,180 (1.86%) and 2,181 (1.86%) were event records.

There were three machine learning algorithms considered and the best candidate model from each algorithm was used in the ensemble model, specific model properties are provided in **Table S7**.

The gradient boosting model gave the highest Gini coefficient of 0.651, AUC of 82.5%, the neural network had the lowest Gini coefficient, 0.604, AUC of 80.2%. The receiver operating curves (**Figure S2**) show that the models are comparable across the three sample datasets, suggesting that the models generalise well to data not used in the training of the model. Goodness of fit statistics are given in **Table S8.**

### Net benefit

Classification of the expected outcome is based on the probability estimated by the model. Upon implementation, a probability cutoff needs to be applied to classify an observation and trigger an action if the observation is classified to be the outcome of interest. The cutoff is based on a balance of sensitivity and specificity and is driven by external factors(2). Net benefit provides a method of measuring the overall advantage gained from deploying a statistical model compared to not using it, in terms of improved decision-making. Net benefit involves weighing elements such as precision, cost benefit, risk assessment, and scalability. The champion model was applied to all people in the OAT cohort for each month in 2017, to calculate the probability of intentional self-harm/suicide in the 30-day window. There was a very small overlap of records used in the model building stage, TRAINING data, < 0.3%). Model posterior probabilities were adjusted using prior probabilities as described in the section: *Adjustment for separate sampling* in supplementary 1. A threshold probability for classifying highest risk is required as well as the feasibility of the number of people to screen to find at least one person at risk of self-harm/suicide. The measurement, net benefit(3), uses an exchange rate to transform the benefits and harms at each threshold probability to a simple assessment measure to compare across models.

The net benefit, defined as benefit minus (harm multiplied by exchange rate), was derived for a range of threshold probabilities Benefit is the true positive rate, harm is the false positive rate and the exchange rate is the odds of the outcome calculated for each of the threshold probabilities, ranging from 0 to 100%. For the strategy of *treatment for all* net benefit is the equivalent of the population event rate minus the non-event rate multiplied by the exchange rate. The exchange rate is a clinical judgement of the relevant value of detection and harms (misclassification), it is where the expected benefit of treatment is equal to the expected benefit of avoiding treatment. In other words, it is how many people a clinician is comfortable referring for further assessment to prevent one event. For clinical prediction models, the exchange rate is related to the threshold probability to decide whether we expect an event in the next 30 days.

As the exchange rate is equal to the threshold probabilities we are assessing in the predictive model it can be interpreted as the odds of correctly identifying a case. Decision curves plotting net benefit against multiple threshold probabilities were created. The purpose of the decision curve is to measure each strategy, the strategy with the highest net benefit at a particular threshold probability has the highest clinical value. We would prefer the prediction model to have higher net benefit than any alternative strategy across a wide range of probabilities.

### Table S1 Glossary

| Term | Description |
| --- | --- |
| Benefit | True positive rate. |
| Data partitioning | Division of a dataset into three datasets: training, validation and test datasets, stratified by the outcome of interest. |
| Decision tree | An empirical tree represents a segmentation of the data that is created by applying a series of simple rules. |
| Gini coefficient | The Gini coefficient is calculated as twice the area between the ROC curve and the diagonal, or as Gini = 2AUC - 1 |
| Gradient boosting | Gradient boosting is an iterative approach that creates multiple trees where, typically, each tree is based on an independent sample without replacement of the data. |
| Exchange rate | The exchange rate is a clinical judgement of the relevant value of detection and harms (misclassification), it is where the expected benefit of treatment is equal to the expected benefit of avoiding treatment. In other words, it is how many people a clinician is comfortable referring for further assessment to prevent one event. The exchange rate is equal to the threshold probabilities we are assessing in the predictive model and can be interpreted as the odds of correctly identifying a case. |
| Harm | False positive rate. |
| Machine learning | The development and study of [statistical algorithms](https://en.wikipedia.org/wiki/Computational_statistics) that can learn from data and [generalize](https://en.wikipedia.org/wiki/Generalize) to unseen data, and thus perform tasks without explicit instructions. |
| Net benefit | *benefit* minus (*harm* multiplied by *exchange rate)*  Strategy of “treatment for all” net benefit is the equivalent of the population event rate minus the non-event rate multiplied by the exchange rate. |
| Neural network | A neural network is a statistical model that is designed to mimic the biological structures of the human brain. |
| Over sampling (separate) | When we have rare events, all records with the event are kept and a subset of non-events. |
| Posterior probabilities | The probability from the model adjusted for over sampling using the formula $\hat{p}_{i}= \frac{\hat{p}_{i}^{*}\rho_{0} \pi_{1}}{\left( 1-\hat{p}_{i}^{*} \right) \rho_{1}\pi_{0}+ \hat{p}_{i}^{*} \rho_{0} \pi_{1}}$ , where model posterior probabilities, $\hat{p}$*_i_*^*^, were adjusted using prior probabilities, π*_i_*, that represent the proportion of the cohort experiencing the outcome (*i*=1) and non-outcome, (*i*=0), ρ_i_ represents the proportion in the training dataset experiencing the outcome *i*. |
| Prior probabilities | The proportion of primary and secondary outcome in the population. |
| Pruning | A technique to optimise complexity and reduce overfitting, by choosing a sub-tree that fits the validation data best. The general idea of pruning is to use an independent validation set to optimize a statistic that summarizes model performance. |
| Regression | A logistic regression attempts to predict the value of a binary or nominal response variable. A logistic regression analysis models the natural logarithm of the odds ratio as a linear combination of the explanatory variables. |
| Shapley values(4) | A prediction can be explained by assuming that each feature value of the instance is a “player” in a game where the prediction is the payout. Shapley values – a method from coalitional game theory – tells us how to fairly distribute the “payout” among the features. In this study the ‘game’ is being able to predict self-harm within a 30-day window for an observation in the dataset. The ‘gain’ is the actual prediction minus the average for all observations. The players are the feature values of each observation. The features work together to achieve the prediction and the goal is to explain the difference between the actual prediction and the average, which feature was most influential. This can differ per observation. The Shapley value is the average marginal contribution of a feature. |
| [SHAP](https://github.com/slundberg/shap) (SHapley Additive exPlanations)(5) | SHAP is an alternative to the Shapley values variables is a method to explain individual predictions, and measures the impact of variables considering the interaction with other variables. Each variable contributes to the final score. It is possible that a slight change in one variable could change the score a lot, while changing another hardly contributes at all on that specific participant. SHAP values calculate the importance of a feature by comparing what a model predicts with and without the feature. The direction of the Shapley measure shows whether it positively or negatively impacts the prediction with the magnitude showing how strong the impact. |
|  |  |
| Test | A holdout dataset used to compare models not used in model building. |
| Threshold probability | The threshold determines the minimum probability required for a positive prediction. |
| Training | The dataset used for model building. |
| Validation | The dataset used to tune the models and prevent overfitting. |
| Variable clustering | Variable clustering removes collinearity, decreases variable redundancy, and helps reveal the underlying structure of the input variables in a data set. For each cluster that contains more than one variable, the variable (or variables) that contribute the most to the variation in that cluster is chosen as the representative variable. |

### Table S2 Self-harm and suicide ICD-10, 9 and SNOMED codes

| **Clinical codeset** | **Cause of death records** |
| --- | --- |
| ICD10 | - Intentional poisoning (X60-X66 and X68-X69), - Violent (X67, X70-X75, and X80-X82), - Cutting or piercing (X78), and - Other (X76-X77, X79, X83-X84, Y870) |
|  | **Self harm – Hospitalisations** |
| ICD10-AM | - Intentional poisoning (X60-X66 and X68-X69), - Violent (X67, X70-X75, and X80-X82), - Cutting or piercing (X78), and - Other (X76-X77, X79, X83-X84, Y87.0) |
|  | **Self harm – Emergency department presentations** |
| ICD10-AM | - Intentional poisoning (X60-X66 and X68-X69), - Violent (X67, X70-X75, and X80-X82), - Cutting or piercing (X78), and - Other (X76-X77, X79, X83-X84, Y87.0) |
| SNOMED-CT (and descendants) | - 276853009, - 418420002, - 248073004, - 1149224003, - 410061008 |
| ICD 9 | - E950-E959 |

| Table S3 Predictor definitions | | |
| --- | --- | --- |
| Predictor | Description | Allowable entries |
| Sex | Sex of the person | Male  Female  Unknown |
| Indigenous_status | Aboriginal and/ or Torres Strait Islander status |  |
| MYBirth | Month and Year of DOB | (mmyyyy) |
| Remoteness | ARIA index based on the postcode of ‘person’s last residence postcode | 1=Major Cities of Australia  2=Inner Regional Australia  3=Outer Regional Australia  4=Remote Australia  5=Very Remote Australia |
| SEIFA | SEIFA index of person’s last residence postcode |  |
| Calendar_year | Year of the cohort start date  Eg: year(01Jan2005)=2005 |  |
| NumED_12mon  NumED _6mon  NumED _3mon | Number of ED presentation in the last 12/6/3 months (12/6/3 months prior to cohort_start_date)?  (excludes the observations for “Issue of repeat prescription”) | 1=Yes  0=No |
| flag_APDC_dep_opi_12  flag_APDC_dep_opi_6  flag_APDC_dep_opi_3 | Is there a hospital admission in the last 12/6/3 months (12/6/3 months prior to cohort_start_date) for Opioid disorder? | 1=Yes  0=No |
| Flag_APDC_dep_oth_12  flag_APDC_dep_oth_6  flag_APDC_dep_oth_3 | Is there a hospital admission in the last 12/6/3 months (12/6/3 months prior to cohort_start_date) for other drug disorder? | 1=Yes  0=No |
| flag_APDC_depanx_12  flag_APDC_depanx_6  flag_APDC_depanx_6 | Is there a hospital admission in the last 12/6/3 months (12/6/3 months prior to cohort_start_date) for depression/anxiety? | 1=Yes  0=No |
| flag_APDC_psyc_12  flag_APDC_psyc_6  flag_APDC_psyc_3 | Is there a hospital admission in the last 12/6/3 months (12/6/3 months prior to cohort_start_date) for psychosis? | 1=Yes  0=No |
| flag_APDC_unk_pois_12  flag_APDC_unk_pois_6  flag_APDC_unk_pois_3 | Is there a hospital admission in the last 12/6/3 months (12/6/3 months prior to cohort_start_date) for poisoning- (intention -unknown)? | 1=Yes  0=No |
| flag_APDC_dep_stim_12  flag_APDC_dep_stim_6  flag_APDC_dep_stim_3 | Is there a hospital admission in the last 12/6/3 months (12/6/3 months prior to cohort_start_date) for stimulants disorder? | 1=Yes  0=No |
| flag_APDC_idea_12  flag_APDC_idea_6  flag_APDC_idea_3 | Is there a hospital admission in the last 12/6/3 months (12/6/3 months prior to cohort_start_date) for suicidal ideation? | 1=Yes  0=No |
| flag_ APDC_dep_alc_12  flag_ APDC_dep_alc_6  flag_ APDC_dep_alc_3 | Is there a hospital admission in the last 12/6/3 months (12/6/3 months prior to cohort_start_date) for alcohol disorder? | 1=Yes  0=No |
| flag_APDC_cpain_12  flag_APDC_cpain_6  flag_APDC_cpain_3 | Is there a hospital admission in the last 12/6/3 months (12 /6/3 months prior to cohort_start_date) for chronic pain? | 1=Yes  0=No |
| flag_APDC_persondisorder_12  flag_APDC_persondisorder_3  flag_APDC_persondisorder_6 | Is there a hospital admission in the last 12/6/3 months (12/6/3 months prior to cohort_start_date) for person disorder? | 1=Yes  0=No |
| flag_APDC_acc_od_12  flag_APDC_acc_od_6  flag_APDC_acc_od_3 | Is there a hospital admission in the last 12/6/3 months (12/6/3 months prior to cohort_start_date) for overdose –(accidental)? | 1=Yes  0=No |
| flag_APDC_si_inj_12  flag_APDC_si_inj_6  flag_APDC_si_inj_3 | Is there a hospital admission in the last 12/6/3 months (12/6/3 months prior to cohort_start_date) for self-inflicted injury)? | 1=Yes  0=No |
| flag_APDC_dep_sed_12  flag_APDC_dep_sed_6  flag_APDC_dep_sed_3 | Is there a hospital admission in the last 12/6/3 months (12/6/3 months prior to cohort_start_date) for sedative disorder)? | 1=Yes  0=No |
| flag_APDC_home_12  flag_APDC_home_6  flag_APDC_home_3 | Is there a hospital admission in the last 12/6/3 months (12 months prior to cohort_start_date) for homelessness)? | 1=Yes  0=No |
| flag_APDC_ho_12  flag_APDC_ho_6  flag_APDC_ho_6 | Is there a hospital admission in the last 12/6/3 months (12/6/3months prior to cohort_start_date) for history of self-harm/ suicide)? | 1=Yes  0=No |
| flag_APDC_acc_pois_12  flag_APDC_acc_pois_6  flag_APDC_acc_pois_3 | Is there a hospital admission in the last 12/6/3 months (12/6/3 months prior to cohort_start_date) for poisoning (accidental)? | 1=Yes  0=No |
| flag_APDC_ptsd_12  flag_APDC_ptsd_6  flag_APDC_ptsd_3 | Is there a hospital admission in the last 12/6/3 months (12/6/3 months prior to cohort_start_date) for PTSD? | 1=Yes  0=No |
| flag_APDC_bpd_12  flag_APDC_bpd_6  flag_APDC_bpd_3 | Is there a hospital admission in the last 12/6/3 months (12/6/3 months prior to cohort_start_date) for BPD? | 1=Yes  0=No |
| flag_APDC_irrit_12  flag_APDC_irrit_6  flag_APDC_irrit_3 | Is there a hospital admission in the last 12/6/3 months (12/6/3 months prior to cohort_start_date) for irritability/anger? | 1=Yes  0=No |
| flag_APDC_dep_coc_12  flag_APDC_dep_coc_6  flag_APDC_dep_coc_6 | Is there a hospital admission in the last 12/6/3 months (1/6/32 months prior to cohort_start_date) for cocaine disorder? | 1=Yes  0=No |
| flag_APDC_int_pois_12  flag_APDC_int_pois_6  flag_APDC_int_pois_3 | Is there a hospital admission in the last 12/6/3 months (12/6/3 months prior to cohort_start_date) for poisoning (intentional)? | 1=Yes  0=No |
| flag_APDC_unk_inj_12  flag_APDC_unk_inj_6  flag_APDC_unk_inj_3 | Is there a hospital admission in the last 12/6/3 months (12/6/3 months prior to cohort_start_date) for unknown injury? | 1=Yes  0=No |
| flag_APDC_int_od_12  flag_APDC_int_od_6  flag_APDC_int_od_3 | Is there a hospital admission in the last 12/6/3 months (12/6/3 months prior to cohort_start_date) for an overdose (intentional )? | 1=Yes  0=No |
| flag_APDC_cabuse_12  flag_APDC_cabuse_6  flag_APDC_cabuse_3 | Is there a hospital admission in the last 12/6/3 months (12/6/3 months prior to cohort_start_date) for history of child abuse? | 1=Yes  0=No |
| flag_ED_dep_opi_12  flag_ED_dep_opi_6  flag_ED_dep_opi_3 | Is there an ED presentation in the last 12/6/3 months (12/6/3 months prior to cohort_start_date) for Opioid disorder? | 1=Yes  0=No |
| flag_ED_dep_oth_12  flag_ED_dep_oth_6  flag_ED_dep_oth_3 | Is there an ED presentation in the last 12/6/3 months (12/6/3 months prior to cohort_start_date) for other drug disorder? | 1=Yes  0=No |
| flag_ED_depanx_12  flag_ED_depanx_6  flag_ED_depanx_6 | Is there an ED presentation in the last 12/6/3 months (12/6/3 months prior to cohort_start_date) for depression/anxiety? | 1=Yes  0=No |
| flag_ED_psyc_12  flag_ED_psyc_6  flag_ED_psyc_3 | Is there an ED presentation in the last 12/6/3 months (12/6/3 months prior to cohort_start_date) for psychosis? | 1=Yes  0=No |
| flag_ED_unk_pois_12  flag_ED_unk_pois_6  flag_ED_unk_pois_3 | Is there an ED presentation in the last 12/6/3 months (12/6/3 months prior to cohort_start_date) for poisoning- (intention- unknown)? | 1=Yes  0=No |
| flag_ED_unk_od_12  flag_ED_unk_od_6  flag_ED_unk_od_3 | Is there an ED presentation in the last 12/6/3 months (12/6/3 months prior to cohort_start_date) for overdose- (intention unknown)? | 1=Yes  0=No |
| flag_ED_dep_stim_12  flag_ED_dep_stim _6  flag_ED_dep_stim _3 | Is an ED presentation in the last 12/6/3 months (12/6/3 months prior to cohort_start_date) for stimulants disorder)? | 1=Yes  0=No |
| flag_ED_idea_12  flag_ED_idea_6  flag_ED_idea_3 | Is there an ED presentation in the last 12/6/3 months (12/6/3 months prior to cohort_start_date) for suicidal ideation)? | 1=Yes  0=No |
| flag_ED_dep_alc_12  flag_ED_dep_alc_6  flag_ED_dep_alc_3 | Is there an ED presentation in the last 12/6/3 months (12/6/3 months prior to cohort_start_date) for alcohol disorder)? | 1=Yes  0=No |
| flag_ED_cpain_12  flag_ED_cpain_6  flag_ED_cpain_3 | Is there an ED presentation in the last 12/6/3 months (12 /6/3 months prior to cohort_start_date) for chronic pain)? | 1=Yes  0=No |
| flag_ED_persondisorder_12  flag_ED_persondisorder_3  flag_ED_persondisorder_6 | Is there an ED presentation in the last 12/6/3 months (12/6/3 months prior to cohort_start_date) for person disorder)? | 1=Yes  0=No |
| flag_ED_acc_od_12  flag_ED_acc_od_6  flag_ED_acc_od_3 | Is there an ED presentation in the last 12/6/3 months (12/6/3 months prior to cohort_start_date) for overdose ( accidental)? | 1=Yes  0=No |
| flag_ED_si_inj_12  flag_ED_si_inj_6  flag_ED_si_inj_3 | Is there an ED presentation in the last 12/6/3 months (12/6/3 months prior to cohort_start_date) for self-injury)? | 1=Yes  0=No |
| flag_ED_dep_sed_12  flag_ED_dep_sed_6  flag_ED_dep_sed_3 | Is there an ED presentation in the last 12/6/3 months (12/6/3 months prior to cohort_start_date) for sedative disorder)? | 1=Yes  0=No |
| flag_ED_home_12  flag_ED_home_6  flag_ED_home_3 | Is there an ED presentation in the last 12/6/3 months (12 months prior to cohort_start_date) for homelessness)? | 1=Yes  0=No |
| flag_ED_ho_12  flag_ED_ho_6  flag_ED_ho_6 | Is there an ED presentation in the last 12/6/3 months (12/6/3months prior to cohort_start_date) for history of self-harm/ suicide)? | 1=Yes  0=No |
| flag_ED_acc_pois_12  flag_ED_acc_pois_6  flag_ED_acc_pois_3 | Is there an ED presentation in the last 12/6/3 months (12/6/3 months prior to cohort_start_date) for poisoning (accidental)? | 1=Yes  0=No |
| flag_ED _ptsd_12  flag_ED_ptsd_6  flag_ED_ptsd_3 | Is there an ED presentation in the last 12/6/3 months (12/6/3 months prior to cohort_start_date) for PTSD? | 1=Yes  0=No |
| flag_ED_bpd_12  flag_ED_bpd_6  flag_ED_bpd_3 | Is there an ED presentation in the last 12/6/3 months (12/6/3 months prior to cohort_start_date) for BPD? | 1=Yes  0=No |
| flag_ED_irrit_12  flag_ED_irrit_6  flag_ED_irrit_3 | Is there an ED presentation in the last 12/6/3 months (12/6/3 months prior to cohort_start_date) for irritability-anger? | 1=Yes  0=No |
| flag_ED_dep_coc_12  flag_ED_dep_coc_6  flag_ED_dep_coc_6 | Is there an ED presentation in the last 12/6/3 months (1/6/32 months prior to cohort_start_date) for cocaine disorder? | 1=Yes  0=No |
| flag_ED_int_pois_12  flag_ED_int_pois_6  flag_ED_int_pois_3 | Is there an ED presentation in the last 12/6/3 months (12/6/3 months prior to cohort_start_date) for poisoning (intentional)? | 1=Yes  0=No |
| flag_ED_unk_inj_12  flag_ED_unk_inj_6  flag_ED_unk_inj_3 | Is there an ED presentation in the last 12/6/3 months (12/6/3 months prior to cohort_start_date) for unknown injury)? | 1=Yes  0=No |
| flag_ED_int_od_12  flag_ED_int_od_6  flag_ED_int_od_3 | Is there an ED presentation in the last 12/6/3 months (12/6/3 months prior to cohort_start_date) for poisoning (intentional)? | 1=Yes  0=No |
| flag_ED_cabuse_12  flag_ED_cabuse_6  flag_ED_cabuse_3 | Is there an ED presentation in the last 12/6/3 months (12/6/3 months prior to cohort_start_date) for history of child abuse? | 1=Yes  0=No |
| Flag_Procedure_12mon  Flag_Procedure_6mon  Flag_Procedure_3mon | Is there a procedure performed as an admitted patient in the last 12/6/3 months (12/6/3 months prior to cohort_start_date)? | 1=Yes  0=No |
| flag_MHAmb_dep_opi_12  flag_MHAmb_dep_opi_6  flag_MHAmb_dep_opi_3 | Is there MH ambulatory presentation in the last 12/6/3 months (12/6/3 months prior to cohort_start_date) for Opioid disorder? | 1=Yes  0=No |
| flag_MHAmb_dep_oth_12  flag_MHAmb_dep_oth_6  flag_MHAmb_dep_oth_3 | Is there an ED presentation in the last 12/6/3 months (12/6/3 months prior to cohort_start_date) for other drug disorder? | 1=Yes  0=No |
| flag_MHAmb_depanx_12  flag_MHAmb_depanx_6  flag_MHAmb_depanx_6 | Is there an ED presentation in the last 12/6/3 months (12/6/3 months prior to cohort_start_date) for depression/anxiety? | 1=Yes  0=No |
| flag_ MHAmb_psyc_12  flag_ MHAmb_psyc_6  flag_ MHAmb_psyc_3 | Is there an ED presentation in the last 12/6/3 months (12/6/3 months prior to cohort_start_date) for psychosis? | 1=Yes  0=No |
| flag_MHAmb_dep_stim_12  flag_MHAmb_dep_stim 6  flag_MHAmb_dep_stim_3 | Is an ED presentation in the last 12/6/3 months (12/6/3 months prior to cohort_start_date) for stimulants disorder)? | 1=Yes  0=No |
| flag_MHAmb_idea_12  flag_MHAmb_idea_6  flag_MHAmb_idea_3 | Is there an ED presentation in the last 12/6/3 months (12/6/3 months prior to cohort_start_date) for suicidal ideation)? | 1=Yes  0=No |
| flag_MHAmb_dep_alc_12  flag_MHAmb_dep_alc_6  flag_MHAmb_dep_alc_3 | Is there an ED presentation in the last 12/6/3 months (12/6/3 months prior to cohort_start_date) for alcohol disorder)? | 1=Yes  0=No |
| flag_MHAmb_persondisorder_12  flag_MHAmb_persondisorder_3  flag_MHAmb_persondisorder_6 | Is there an ED presentation in the last 12/6/3 months (12/6/3 months prior to cohort_start_date) for person disorder)? | 1=Yes  0=No |
| flag_MHAmb_si_inj_12  flag_MHAmb_si_inj_6  flag_MHAmb_si_inj_3 | Is there an ED presentation in the last 12/6/3 months (12/6/3 months prior to cohort_start_date) for self-inflicted injury)? | 1=Yes  0=No |
| flag_MHAmb_dep_sed_12  flag_MHAmb_dep_sed_6  flag_MHAmb_dep_sed_3 | Is there an ED presentation in the last 12/6/3 months (12/6/3 months prior to cohort_start_date) for sedative disorder? | 1=Yes  0=No |
| flag_MHAmb_ptsd_12  flag_MHAmb_ptsd_6  flag_MHAmb_ptsd_3 | Is there an ED presentation in the last 12/6/3 months (12/6/3 months prior to cohort_start_date) for PTSD? | 1=Yes  0=No |
| flag_MHAmb_dep_coc_12  flag_MHAmb_dep_coc_6  flag_MHAmb_dep_coc_6 | Is there an ED presentation in the last 12/6/3 months (1/6/32 months prior to cohort_start_date) for cocaine disorder? | 1=Yes  0=No |
| Elapsed_time | Time since the initial OAT encounter  From initial OAT start date to current_cohort_start date (In days) |  |
| Flag_Change_med | Is there a medication change in last 30 days? | 1=Yes  0=No |
| Flag_change_prescriber | Is there a prescriber change in last 30 days? | 1=Yes  0=No |
| Flag_change_DosingPointSector | Is there a dosing point sector change in last 30 days? | 1=Yes  0=No |
| Flag_change_DosingPointType | Is there a dosing point type change in last 30 days? | 1=Yes  0=No |
| Num_prescriber_12mon | Number of prescriber’s last 12 months |  |
| Num_prescriber_6mon | Number of prescriber’s last 6 months |  |
| Num_prescriber_3mon | Number of prescriber’s last 3 months |  |
| Num_OAT_12mon | Number of OAT episodes last 12 months |  |
| Num_OAT_6mon | Number of OAT episodes last 6 months |  |
| Num_OAT_3mon | Number of OAT episodes last 3 months |  |
| Num_ViolentCharges_12mon | Number of violent charges last 12 months (regardless of outcome)  earliest_first_appearance_date  between cohort_start-1 and 12 months before cohort_start date -1  if earliest_first_appearance_date is not available use finalisation_date |  |
| Num_ ViolentCharges _6mon | Number of violent charges last 6 months (regardless of outcome) |  |
| Num_ ViolentCharges _3mon | Number of violent charges last 3 months(regardless of outcome) |  |
| Num_NonViolentCharges_12mon | Number of non-violent charges last 12 months |  |
| Num_NonViolentCharges _6mon | Number of non-violent charges last 6 months |  |
| Num_NonViolentCharges _3mon | Number of non-violent charges last 3 months |  |
| Num_ViolentConvictions_12mon | Number of violent charges last 12 months (regardless of outcome) |  |
| Num_ ViolentConvictions _6mon | Number of violent charges last 6 months (regardless of outcome) |  |
| Num_ ViolentConvictions _3mon | Number of violent charges last 3 months(regardless of outcome) |  |
| Num_NonViolentConvictions_12mon | Number of non-violent charges last 12 months |  |
| Num_NonViolentConvictions _6mon | Number of non-violent charges last 6 months |  |
| Num_ NonViolentConvictions _3mon | Number of non-violent charges last 3 months |  |
| Recent_Incarceration_3M | Released from incarceration last 3 month |  |
| Recent_Incarceration_6M | Released from incarceration last 6 month |  |
| Recent_Incarceration_12M | Released from incarceration last 12 month |  |
| OAT_30 | Number of days in treatment in the last 30 days prior to cohort_start |  |
| OAT_60 | Number of days in treatment in the last 60 days prior to cohort_start |  |
| OAT_90 | Number of days in treatment in the last 90 days prior to cohort_start |  |

| Table S4 ICD and SNOMED codes used for health service utilisation | | | |
| --- | --- | --- | --- |
| **Predictor at 3, 6 and 12 months** | **Code type** | **Code** | **Description** |
| flag_APDC_acc_od  flag_ED_acc_od | ICD-10-AM | X40 | Accidental poisoning by and exposure to nonopioid analgesics, antipyretics and antirheumatics |
|  | ICD-10-AM | X41 | Accidental poisoning by and exposure to antiepileptic, sedative-hypnotic, antiparkinsonism and psych |
|  | ICD-10-AM | X42 | Accidental poisoning by and exposure to narcotics and psychodysleptics [hallucinogens], not elsewher |
|  | ICD-10-AM | X43 | Accidental poisoning by and exposure to other drugs acting on the autonomic nervous system |
|  | ICD-10-AM | X44 | Accidental poisoning by and exposure to other and unspecified drugs, medicaments and biological subs |
|  | ICD-10-AM | X45 | Accidental poisoning by and exposure to alcohol |
|  | ICD-10-AM | X46 | Accidental poisoning by and exposure to organic solvents and halogenated hydrocarbons and their vapo |
|  | SNOMED CT | Descendant codes of 1149222004\|Overdose\| containing "Accidental" | Accidental overdose |
|  | SNOMED CT | 59369008 | Accidental drug overdose (disorder) |
| flag_APDC_acc_pois  flag_ED_acc_pois | ICD-10-AM | X47 | Accidental poisoning by and exposure to other gases and vapours |
|  | ICD-10-AM | X48 | Accidental poisoning by and exposure to pesticides |
|  | ICD-10-AM | X49 | Accidental poisoning by and exposure to other and unspecified chemicals and noxious substances |
|  | SNOMED CT | Descendant codes of 7895008\|Drug toxicity\| containing "Accidental" | Accidental 5-aminosalicylic acid poisoning |
| flag_APDC_bpd  flag_ED_bpd | ICD-10-AM | F60.3 | Emotionally unstable personality disorder |
|  | ICD-9 | 301.3 | Explosive personality disorder |
|  | ICD-9 | 301.83 | Borderline personality disorder |
|  | SNOMED CT | 20010003 and descendants\|Borderline personality disorder | Borderline personality disorder |
| flag_APDC_cabuse  flag_ED_cabuse | ICD-10-AM | T74.9 | Maltreatment syndrome, unspecified |
|  | ICD-10-AM | Z62.9 | Problem related to upbringing, unspecified |
|  | ICD-10-AM | Z62.8 | Other specified problems related to upbringing |
|  | ICD-10-AM | Z62.5 | Other problems related to neglect in upbringing |
|  | SNOMED CT | 288541000119107 | Problem related to upbringing |
| flag_APDC_cpain  flag_ED_cpain | ICD-10-AM | R52.1 | Chronic intractable pain |
|  | ICD-10-AM | R52.2 | Other chronic pain |
|  | SNOMED CT | 82423001 and its descendants\|Chronic pain\| | Abdominal cutaneous nerve entrapment syndrome |
| flag_ APDC_dep_alc  flag_ ED_dep_alc | ICD-10-AM | F10 | Mental and behavioural disorders due to use of alcohol |
|  | ICD-9 | 291 | Alcohol-induced mental disorders |
|  | SNOMED CT | 191816009 and its descendants\|Drug dependence\| | Alcohol dependence |
|  | SNOMED CT | 15167005 | Alcohol abuse |
|  | SNOMED CT | 191476005 | Alcohol withdrawal hallucinosis |
|  | SNOMED CT | 191480000 | Alcohol withdrawal syndrome |
|  | SNOMED CT | 284591009 | Persistent alcohol abuse |
|  | SNOMED CT | 29212009 | Alcohol-induced organic mental disorder |
|  | SNOMED CT | 42344001 | Alcohol-induced psychosis |
|  | SNOMED CT | 53936005 | Alcohol-induced mood disorder |
|  | SNOMED CT | 66590003 | Alcohol dependence |
|  | SNOMED CT | 7200002 | Alcoholism |
|  | SNOMED CT | 85561006 | Uncomplicated alcohol withdrawal |
|  | SNOMED CT | 8635005 | Alcohol withdrawal delirium |
| flag_APDC_dep_coc  flag_ED_dep_coc | ICD-10-AM | F14 | Mental and behavioural disorders due to use of cocaine |
|  | ICD-9 | 304.2 | Cocaine dependence |
|  | SNOMED CT | 31956009 | Cocaine dependence |
|  | SNOMED CT | 191833002 | Cocaine dependence in remission |
|  | SNOMED CT | 191831000 | Cocaine dependence, continuous |
|  | SNOMED CT | 191832007 | Cocaine dependence, episodic |
|  | SNOMED CT | 27956007 | Cocaine intoxication |
|  | SNOMED CT | 46975003 | Cocaine-induced organic mental disorder |
|  | SNOMED CT | 80868005 | Cocaine withdrawal |
| flag_APDC_dep_opi  flag_ED_dep_opi  flag_MHAmb_dep_opi | ICD-10-AM | F11 | Mental and behavioural disorders due to use of opioids |
|  | ICD-9 | 304 | Opioid type dependence |
|  | ICD-9 | 304.7 | Combinations of opioid type drug with any other drug dependence |
|  | SNOMED CT | 703845008 | Buprenorphine dependence |
|  | SNOMED CT | 191865004 | Combined opioid with other drug dependence |
|  | SNOMED CT | 191869005 | Combined opioid with other drug dependence in remission |
|  | SNOMED CT | 191867007 | Combined opioid with other drug dependence, continuous |
|  | SNOMED CT | 191868002 | Combined opioid with other drug dependence, episodic |
|  | SNOMED CT | 191819002 | Continuous opioid dependence |
|  | SNOMED CT | 95919007 | Dependence on corticoids |
|  | SNOMED CT | 191820008 | Episodic opioid dependence |
|  | SNOMED CT | 1607669100 | Episodic polysubstance dependence |
|  | SNOMED CT | 231477003 | Heroin dependence |
|  | SNOMED CT | 231478008 | Methadone dependence |
|  | SNOMED CT | 231479000 | Morphine dependence |
|  | SNOMED CT | 75544000 | Opioid dependence |
|  | SNOMED CT | 191821007 | Opioid dependence in remission |
|  | SNOMED CT | 1081000119 | Opioid dependence, on agonist therapy |
|  | SNOMED CT | 231480002 | Opium dependence |
|  | SNOMED CT | 14784000 | Opioid-induced organic mental disorder |
|  | SNOMED CT | 87132004 | Opioid withdrawal |
| flag_apdc_dep_oth  flag_ed_dep_oth  flag_MHAmb_dep_oth | ICD-10-AM | F12 | Mental and behavioural disorders due to use of cannabinoids |
|  | ICD-10-AM | F17 | Mental and behavioural disorders due to use of tobacco |
|  | ICD-10-AM | F18 | Mental and behavioural disorders due to use of volatile solvents |
|  | ICD-10-AM | F19 | Mental and behavioural disorders due to multiple drug use and use of other psychoactive substances |
|  | ICD-9 | 304.6 | Other specified drug dependence |
|  | ICD-9 | 304.8 | Combinations of drug dependence excluding opioid type drug |
|  | ICD-9 | 304.3 | Cannabis dependence |
|  | ICD-9 | 304.9 | Unspecified drug dependence |
|  | ICD-9 | 292 | Drug-induced mental disorders |
|  | SNOMED CT | 231467000 | Absinthe addiction |
|  | SNOMED CT | 1382351000 | Aerosol inhalation dependence |
|  | SNOMED CT | 231470001 | Anxiolytic dependence |
|  | SNOMED CT | 1231159006 | Anxiolytic dependence in remission |
|  | SNOMED CT | 1231163004 | Anxiolytic dependence, continuous |
|  | SNOMED CT | 1231161002 | Anxiolytic dependence, episodic |
|  | SNOMED CT | 1382441000 | Butane inhalation dependence |
|  | SNOMED CT | 85005007 | Cannabis dependence |
|  | SNOMED CT | 191839003 | Cannabis dependence in remission |
|  | SNOMED CT | 191837001 | Cannabis dependence, continuous |
|  | SNOMED CT | 191838006 | Cannabis dependence, episodic |
|  | SNOMED CT | 817962007 | Chlordiazepoxide dependence |
|  | SNOMED CT | 191813001 | Chronic alcoholism in remission |
|  | SNOMED CT | 429299000 | Combined drug dependence, continuous |
|  | SNOMED CT | 191873008 | Combined drug dependence, excluding opioid, continuous |
|  | SNOMED CT | 191874002 | Combined drug dependence, excluding opioid, episodic |
|  | SNOMED CT | 191875001 | Combined drug dependence, excluding opioid, in remission |
|  | SNOMED CT | 191871005 | Combined drug dependence, excluding opioids |
|  | SNOMED CT | 191816009 | Drug dependence |
|  | SNOMED CT | 199252002 | Drug dependence during pregnancy - baby delivered |
|  | SNOMED CT | 199254001 | Drug dependence during pregnancy - baby not yet delivered |
|  | SNOMED CT | 267206008 | Drug dependence during pregnancy, childbirth and the puerperium |
|  | SNOMED CT | 1075516100 | Drug dependence in childbirth |
|  | SNOMED CT | 34150001 | Drug dependence in mother complicating pregnancy, childbirth AND/OR puerperium |
|  | SNOMED CT | 1461000119 | Drug dependence in remission |
|  | SNOMED CT | 199253007 | Drug dependence in the puerperium - baby delivered |
|  | SNOMED CT | 199255000 | Drug dependence in the puerperium - baby delivered during previous episode of care |
|  | SNOMED CT | 1534910001 | Drug dependence, continuous |
|  | SNOMED CT | 1535010001 | Drug dependence, episodic |
|  | SNOMED CT | 426001001 | Fentanyl dependence |
|  | SNOMED CT | 191853003 | Glue sniffing dependence |
|  | SNOMED CT | 191857002 | Glue sniffing dependence in remission |
|  | SNOMED CT | 191855005 | Glue sniffing dependence, continuous |
|  | SNOMED CT | 191856006 | Glue sniffing dependence, episodic |
|  | SNOMED CT | 5002000 | Inhalant dependence |
|  | SNOMED CT | 8639100011 | Inhalant dependence, continuous |
|  | SNOMED CT | 8640100011 | Inhalant dependence, episodic |
|  | SNOMED CT | 231468005 | Lysergic acid diethylamide dependence |
|  | SNOMED CT | 231469002 | Mescaline dependence |
|  | SNOMED CT | 56294008 | Nicotine dependence |
|  | SNOMED CT | 836439001 | Nicotine dependence in remission |
|  | SNOMED CT | 1382421000 | Paint sniffing dependence |
|  | SNOMED CT | 1382391000 | Petrol sniffing dependence |
|  | SNOMED CT | 51339003 | Polysubstance dependence |
|  | SNOMED CT | 237228001 | Pregnancy and drug dependence |
|  | SNOMED CT | 737337007 | Synthetic cannabinoid dependence |
|  | SNOMED CT | 762505006 | Synthetic cathinone dependence |
|  | SNOMED CT | 95918004 | Therapeutic drug dependence |
|  | SNOMED CT | 1607717100 | Tobacco dependence caused by chewing tobacco |
|  | SNOMED CT | 1607709100 | Tobacco dependence caused by cigarettes |
|  | SNOMED CT | 1607705100 | Tobacco dependence caused by cigarettes in remission |
|  | SNOMED CT | 191889006 | Tobacco dependence in remission |
|  | SNOMED CT | 89765005 | Tobacco dependence syndrome |
|  | SNOMED CT | 191887008 | Tobacco dependence, continuous |
|  | SNOMED CT | 191888003 | Tobacco dependence, episodic |
|  | SNOMED CT | 11061003 | Psychoactive substance use disorder |
|  | SNOMED CT | 11387009 | Psychoactive substance-induced organic mental disorder |
|  | SNOMED CT | 191483003 | Drug-induced psychosis |
|  | SNOMED CT | 191484009 | Drug-induced paranoia or hallucinatory states |
|  | SNOMED CT | 191492000 | Drug-induced delirium |
|  | SNOMED CT | 191494004 | Drug-induced amnestic syndrome |
|  | SNOMED CT | 191495003 | Drug-induced depressive state |
|  | SNOMED CT | 191496002 | Drug-induced personality disorder |
|  | SNOMED CT | 231459008 | Abuse of nonpsychotropic analgesic drugs |
|  | SNOMED CT | 2403008 | Psychoactive substance dependence |
|  | SNOMED CT | 39951001 | Cannabis-induced anxiety disorder |
|  | SNOMED CT | 74934004 | Psychoactive substance-induced withdrawal syndrome |
|  | SNOMED CT | 77355000 | Cannabis-induced organic mental disorder |
|  | SNOMED CT | 90755006 | Nicotine withdrawal |
| flag_APDC_dep_sed  flag_ED_dep_sed  flag_MHAMB_dep_sed | ICD-10-AM | F13 | Mental and behavioural disorders due to use of sedatives or hypnotics |
|  | ICD-9 | 304.1 | Sedative, hypnotic or anxiolytic dependence |
|  | SNOMED CT | 231472009 | Barbiturate dependence |
|  | SNOMED CT | 231473004 | Benzodiazepine dependence |
|  | SNOMED CT | 724715004 | Dependence due to ketamine |
|  | SNOMED CT | 231474005 | Diazepam dependence |
|  | SNOMED CT | 724656006 | Hypnotic dependence |
|  | SNOMED CT | 1231158003 | Hypnotic dependence in remission |
|  | SNOMED CT | 1231162009 | Hypnotic dependence, continuous |
|  | SNOMED CT | 1231160001 | Hypnotic dependence, episodic |
|  | SNOMED CT | 1382451000 | Nitrite inhalation dependence |
|  | SNOMED CT | 1382511000 | Nitrous oxide inhalation dependence |
|  | SNOMED CT | 58727001 | PCP dependence |
|  | SNOMED CT | 425841004 | Phencyclidine dependence in remission |
|  | SNOMED CT | 427327003 | Sedative dependence |
| flag_apdc_dep_stim  flag_ED_dep_stim  flag_MHAMB_dep_stim | ICD-10-AM | F15 | Mental and behavioural disorders due to use of other stimulants, including caffeine |
|  | ICD-10-AM | F16 | Mental and behavioural disorders due to use of hallucinogens |
|  | ICD-9 | 304.4 | Amphetamine and other psychostimulant dependence |
|  | ICD-9 | 304.5 | Hallucinogen dependence |
|  | SNOMED CT | 1231328003 | Amfetamine and/or amfetamine derivative dependence continuous |
|  | SNOMED CT | 1231327008 | Amfetamine and/or amfetamine derivative dependence episodic |
|  | SNOMED CT | 1231325000 | Amfetamine and/or amfetamine derivative dependence in remission |
|  | SNOMED CT | 838527002 | Amfetamine and/or amfetamine derivative drug dependence |
|  | SNOMED CT | 21647008 | Amphetamine dependence |
|  | SNOMED CT | 428219007 | Caffeine dependence |
|  | SNOMED CT | 38247002 | Hallucinogen dependence |
|  | SNOMED CT | 191851001 | Hallucinogen dependence in remission |
|  | SNOMED CT | 191849000 | Hallucinogen dependence, continuous |
|  | SNOMED CT | 191850000 | Hallucinogen dependence, episodic |
|  | SNOMED CT | 426873000 | Methamphetamine dependence |
|  | SNOMED CT | 1231319004 | Methylenedioxymethamphetamine dependence |
|  | SNOMED CT | 2403008 | Psychoactive substance dependence |
|  | SNOMED CT | 275471001 | Psychostimulant dependence |
|  | SNOMED CT | 1231336007 | Psychostimulant dependence continuous |
|  | SNOMED CT | 1231333004 | Psychostimulant dependence episodic |
|  | SNOMED CT | 1231331002 | Psychostimulant dependence in remission |
|  | SNOMED CT | 442406005 | Stimulant dependence |
|  | SNOMED CT | 191928000 | Abuse of antidepressant drug |
|  | SNOMED CT | 78358001 | Amphetamine withdrawal |
|  | SNOMED CT | 83367009 | Amphetamine-induced organic mental disorder |
|  | SNOMED CT | 8837000 | Amphetamine delirium |
| flag_APDC_depanx  flag_ED_depanx  flag_MHAMB_depanx | ICD-10-AM | F32.0 | Mild depressive episode |
|  | ICD-10-AM | F32.1 | Moderate depressive episode |
|  | ICD-10-AM | F32.2 | Severe depressive episode without psychotic symptoms |
|  | ICD-10-AM | F32.8 | Other depressive episodes |
|  | ICD-10-AM | F32.9 | Depressive episode, unspecified |
|  | ICD-10-AM | F33 | Recurrent depressive disorder |
|  | ICD-10-AM | F34.1 | Dysthymia |
|  | ICD-10-AM | F38 | Other mood [affective] disorders |
|  | ICD-10-AM | F39 | Unspecified mood [affective] disorder |
|  | ICD-10-AM | F40 | Phobic anxiety disorders |
|  | ICD-10-AM | F41 | Other anxiety disorders |
|  | ICD-10-AM | F42 | Obsessive-compulsive disorder |
|  | ICD-9 | 296 | Episodic mood disorders |
|  | ICD-9 | 300 | Anxiety, dissociative and somatoform disorders |
|  | ICD-9 | 311 | Depressive disorder, not elsewhere classified |
|  | SNOMED CT | 35489007 and its descendants\|Depression\| | Depression |
|  | SNOMED CT | 48694002 and its descendants\|Anxiety\| | Anxiety |
|  | SNOMED CT | 192080009 | Chronic depression |
|  | SNOMED CT | 197480006 | Anxiety disorder |
|  | SNOMED CT | 231504006 | Mixed anxiety and depressive disorder |
|  | SNOMED CT | 35489007 | Depression |
|  | SNOMED CT | 35919005 | Pervasive developmental disorder |
|  | SNOMED CT | 46206005 | Mood disorder |
|  | SNOMED CT | 83458005 | Agitated depression |
| flag_APDC_ho  flag_ED_ho | ICD-10-AM | Z86.5 | Personal history of other mental and behavioural disorders |
|  | ICD-10-AM | Z91.5 | Personal history of self-harm |
|  | ICD-9 | V15.59 | Personal history of self-harm |
|  | SNOMED CT | 314550003 | H/O: deliberate self harm |
|  | SNOMED CT | 161474000 | H/O: attempted suicide |
| flag_APDC_home | ICD-10-AM | Z59.0 | Homelessness |
|  | ICD-10-AM | Z59.1 | Inadequate housing |
|  | ICD-10-AM | Z59.8 | Other problems related to housing and economic circumstances |
|  | ICD-10-AM | Z59.9 | Problem related to housing and economic circumstances, unspecified |
|  | ICD-9 | V60.0 | Homelessness |
|  | SNOMED CT | 32911000 and its descendants\|Homeless\| | Homeless |
| flag_APDC_idea  flag_ED_idea  flag_MHAMB_idea | ICD-10-AM | R45.8 | Other symptoms and signs involving emotional state |
|  | ICD-9 | V62.84 | Suicidal ideation |
|  | SNOMED CT | 225444004 and its descendants\|Suicide risk\| | Suicide risk |
|  | SNOMED CT | 6471006 and its descendants\|Suicidal thoughts\| | Suicidal thoughts |
|  | SNOMED CT | 41501003 | Suicide risk |
|  | SNOMED CT | 225457007 | Feeling suicidal (finding) |
| flag_APDC_int_od  flag_ED_int_od | ICD-10-AM | X60 | Intentional self-poisoning by and exposure to nonopioid analgesics, antipyretics and antirheumatics |
|  | ICD-10-AM | X61 | Intentional self-poisoning by and exposure to antiepileptic, sedative-hypnotic, antiparkinsonism and |
|  | ICD-10-AM | X62 | Intentional self-poisoning by and exposure to narcotics and psychodysleptics [hallucinogens], not el |
|  | ICD-10-AM | X63 | Intentional self-poisoning by and exposure to other drugs acting on the autonomic nervous system |
|  | ICD-10-AM | X64 | Intentional self-poisoning by and exposure to other and unspecified drugs, medicaments and biologica |
|  | ICD-10-AM | X65 | Intentional self-poisoning by and exposure to alcohol |
|  | ICD-10-AM | X66 | Intentional self-poisoning by and exposure to organic solvents and halogenated hydrocarbons and thei |
|  | ICD-9 | E950 | Suicide and self-inflicted poisoning by solid or liquid substances |
|  | SNOMED CT | Descendants of 1149222004\|Overdose\| containing "Intentional" | Intentional 5-aminosalicylic acid overdose |
|  | SNOMED CT | 59274003 | Intentional drug overdose (disorder) |
|  | SNOMED CT | 242828004 | Intentional opiate analgesic overdose (disorder) |
| flag_APDC_int_pois  flag_ED_int_pois | ICD-10-AM | X67 | Intentional self-poisoning by and exposure to other gases and vapours |
|  | ICD-10-AM | X68 | Intentional self-poisoning by and exposure to pesticides |
|  | ICD-10-AM | X69 | Intentional self-poisoning by and exposure to other and unspecified chemicals and noxious substances |
|  | ICD-9 | E951 | Suicide and self-inflicted poisoning by gases in domestic use |
|  | ICD-9 | E952 | Suicide and self-inflicted poisoning by other gases and vapors |
|  | SNOMED CT | Descendants of 7895008\|Drug toxicity\| containing "Intentional" | Intentional 5-aminosalicylic acid poisoning |
| flag_APDC_irrit  flag_ED_irrit | ICD-10-AM | R45.1 | Restlessness and agitation |
|  | ICD-9 | 307.9 | Restlessness and agitation |
|  | SNOMED CT | 274646000\|Irritability and its descendants | Irritability and anger |
| flag_APDC_persondisorder  flag_ED_persondisorder  flag_MHAMB_persondisorder | ICD-10-AM | F60.0 | Paranoid personality disorder |
|  | ICD-10-AM | F60.1 | Schizoid personality disorder |
|  | ICD-10-AM | F60.2 | Dissocial personality disorder |
|  | ICD-10-AM | F60.4 | Histrionic personality disorder |
|  | ICD-10-AM | F60.5 | Anankastic personality disorder |
|  | ICD-10-AM | F60.6 | Anxious [avoidant] personality disorder |
|  | ICD-10-AM | F60.7 | Dependent personality disorder |
|  | ICD-10-AM | F60.8 | Other specific personality disorders |
|  | ICD-10-AM | F60.9 | Personality disorder, unspecified |
|  | ICD-10-AM | F61 | Mixed and other personality disorders |
|  | ICD-9 | 301 | Paranoid |
|  | ICD-9 | 301.2 | Schizoid |
|  | ICD-9 | 301.9 | Psychopathic |
|  | SNOMED CT | 33449004\|Personality disorder and its descendants | Acute exacerbation of chronic latent schizophrenia |
|  | SNOMED CT | 191667009 | Paranoid disorder |
|  | SNOMED CT | 26665006 | Antisocial personality disorder |
|  | SNOMED CT | 33449004 | Personality disorder |
|  | SNOMED CT | 370143000 | Major depressive disorder |
|  | SNOMED CT | 386820009 | Socialised behaviour disorder |
|  | SNOMED CT | 4306003 | Cluster B personality disorder |
| flag_APDC_psyc  flag_ED_psyc  flag_MHAMB_psyc | ICD-10-AM | F20 | Schizophrenia |
|  | ICD-10-AM | F22 | Persistent delusional disorders |
|  | ICD-10-AM | F23 | Acute and transient psychotic disorders |
|  | ICD-10-AM | F24 | Induced delusional disorder |
|  | ICD-10-AM | F25 | Schizoaffective disorders |
|  | ICD-10-AM | F28 | Other nonorganic psychotic disorders |
|  | ICD-10-AM | F29 | Unspecified nonorganic psychosis |
|  | ICD-10-AM | F30.2 | Mania with psychotic symptoms |
|  | ICD-10-AM | F31.2 | Bipolar affective disorder, current episode manic with psychotic symptoms |
|  | ICD-10-AM | F31.5 | Bipolar affective disorder, current episode severe depression with psychotic symptoms |
|  | ICD-9 | 295 | Schizophrenic disorders |
|  | ICD-9 | 297 | Delusional disorders |
|  | ICD-9 | 298 | Other nonorganic psychoses |
|  | SNOMED CT | 69322001\|Psychotic disorder and its descendants | Acute exacerbation of chronic catatonic schizophrenia |
|  | SNOMED CT | 260994008 | Bipolar |
|  | SNOMED CT | 48500005 | Delusional disorder |
|  | SNOMED CT | 13746004 | Bipolar affective disorder |
|  | SNOMED CT | 1196001 | Chronic bipolar II disorder, most recent episode major depressive |
|  | SNOMED CT | 128293007 | Chronic mental disorder |
|  | SNOMED CT | 13313007 | Mild bipolar disorder |
|  | SNOMED CT | 13746004 | Bipolar disorder |
|  | SNOMED CT | 16506000 | Mixed bipolar I disorder |
|  | SNOMED CT | 191531007 | Acute exacerbation of chronic schizophrenia |
|  | SNOMED CT | 191572009 | Acute exacerbation of chronic schizoaffective schizophrenia |
|  | SNOMED CT | 191590005 | Recurrent manic episodes |
|  | SNOMED CT | 191618007 | Bipolar affective disorder, current episode manic |
|  | SNOMED CT | 191620005 | Bipolar affective disorder, currently manic, mild |
|  | SNOMED CT | 191621009 | Bipolar affective disorder, currently manic, moderate |
|  | SNOMED CT | 191625000 | Bipolar affective disorder, currently manic, in full remission |
|  | SNOMED CT | 191636007 | Mixed bipolar affective disorder |
|  | SNOMED CT | 191677006 | Acute hysterical psychosis |
|  | SNOMED CT | 192362008 | Bipolar affective disorder, current episode mixed |
|  | SNOMED CT | 231487004 | Persistent delusional disorder |
|  | SNOMED CT | 26516009 | Severe mood disorder with psychotic features |
|  | SNOMED CT | 268619003 | Manic disorder, single episode |
|  | SNOMED CT | 268622001 | Chronic paranoid psychosis |
|  | SNOMED CT | 268624000 | Acute paranoid reaction |
|  | SNOMED CT | 278853003 | Acute schizophrenia-like psychotic disorder |
|  | SNOMED CT | 280994000 | Chronic confusional state |
|  | SNOMED CT | 286933003 | Confusional state |
|  | SNOMED CT | 31446002 | Bipolar affective disorder, current episode hypomanic |
|  | SNOMED CT | 31658008 | Chronic paranoid schizophrenia |
|  | SNOMED CT | 371596008 | Bipolar I disorder |
|  | SNOMED CT | 371600003 | Severe bipolar disorder |
|  | SNOMED CT | 48500005 | Delusional disorder |
|  | SNOMED CT | 48937005 | Bipolar II disorder, most recent episode hypomanic |
|  | SNOMED CT | 49468007 | Depressed bipolar I disorder |
|  | SNOMED CT | 5464005 | Brief reactive psychosis |
|  | SNOMED CT | 58214004 | Schizophrenia |
|  | SNOMED CT | 61831009 | Induced psychotic disorder |
|  | SNOMED CT | 64905009 | Paranoid schizophrenia |
|  | SNOMED CT | 68890003 | Schizoaffective disorder |
|  | SNOMED CT | 69322001 | Psychotic disorder |
|  | SNOMED CT | 83746006 | Chronic schizophrenia |
| flag_APDC_ptsd  flag_ED_ptsd  flag_MHAMB_ptsd | ICD-10-AM | F43.1 | Post traumatic stress disorder |
|  | SNOMED CT | 47505003\|Post-traumatic stress disorder and its descendants | Post-traumatic stress disorder |
| flag_APDC_si_inj  flag_ED_si_inj  flag_MHAMB_si_inj | ICD-10-AM | X70 | Intentional self-harm by hanging, strangulation and suffocation |
|  | ICD-10-AM | X71 | Intentional self-harm by drowning and submersion |
|  | ICD-10-AM | X72 | Intentional self-harm by handgun discharge |
|  | ICD-10-AM | X73 | Intentional self-harm by rifle, shotgun and larger firearm discharge |
|  | ICD-10-AM | X74 | Intentional self-harm by other and unspecified firearm discharge |
|  | ICD-10-AM | X75 | Intentional self-harm by explosive material |
|  | ICD-10-AM | X76 | Intentional self-harm by smoke, fire and flames |
|  | ICD-10-AM | X77 | Intentional self-harm by steam, hot vapours and hot objects |
|  | ICD-10-AM | X78 | Intentional self-harm by sharp object |
|  | ICD-10-AM | X79 | Intentional self-harm by blunt object |
|  | ICD-10-AM | X80 | Intentional self-harm by jumping from a high place |
|  | ICD-10-AM | X81 | Intentional self-harm by jumping or lying before moving object |
|  | ICD-10-AM | X82 | Intentional self-harm by crashing of motor vehicle |
|  | ICD-10-AM | X83 | Intentional self-harm by other specified means |
|  | ICD-10-AM | X84 | Intentional self-harm by unspecified means |
|  | ICD-10-AM | Y87 | Sequelae of intentional self-harm, assault and events of undetermined intent |
|  | ICD-9 | E953 | Suicide and self-inflicted injury by hanging strangulation and suffocation |
|  | ICD-9 | E954 | Suicide and self-inflicted injury by submersion [drowning] |
|  | ICD-9 | E955 | Suicide and self-inflicted injury by firearms air guns and explosives |
|  | ICD-9 | E956 | Suicide and self-inflicted injury by cutting and piercing instrument |
|  | ICD-9 | E957 | Suicide and self-inflicted injuries by jumping from high place |
|  | ICD-9 | E958 | Suicide and self-inflicted injury by other and unspecified means |
|  | ICD-9 | E959 | Late effects of self-inflicted injury |
|  | SNOMED CT | 276853009\|Self inflicted injury and its descendants | Self inflicted injury |
|  | SNOMED CT | 418420002\|Intentionally harming self and it's descendants | Intentionally harming self |
|  | SNOMED CT | 248073004\|Cutting self\| and its descendants | Cutting self |
| flag_APDC_unk_inj  flag_ED_unk_inj | ICD-10-AM | Y17 | Poisoning by and exposure to other gases and vapours, undetermined intent |
|  | ICD-10-AM | Y20 | Hanging, strangulation and suffocation, undetermined intent |
|  | ICD-10-AM | Y21 | Drowning and submersion, undetermined intent |
|  | ICD-10-AM | Y22 | Handgun discharge, undetermined intent |
|  | ICD-10-AM | Y23 | Rifle, shotgun and larger firearm discharge, undetermined intent |
|  | ICD-10-AM | Y24 | Other and unspecified firearm discharge, undetermined intent |
|  | ICD-10-AM | Y25 | Contact with explosive material, undetermined intent |
|  | ICD-10-AM | Y26 | Exposure to smoke, fire and flames, undetermined intent |
|  | ICD-10-AM | Y27 | Contact with steam, hot vapours and hot objects, undetermined intent |
|  | ICD-10-AM | Y28 | Contact with sharp object, undetermined intent |
|  | ICD-10-AM | Y29 | Contact with blunt object, undetermined intent |
|  | ICD-10-AM | Y30 | Falling, jumping or pushed from a high place, undetermined intent |
|  | ICD-10-AM | Y31 | Falling, lying or running before or into moving object, undetermined intent |
|  | ICD-10-AM | Y32 | Crashing of motor vehicle, undetermined intent |
|  | ICD-10-AM | Y33 | Other specified events, undetermined intent |
|  | ICD-10-AM | Y34 | Unspecified event, undetermined intent |
| flag_APDC_unk_od flag_ED_unk_od | ICD-10-AM | Y10 | Poisoning by and exposure to nonopioid analgesics, antipyretics and antirheumatics, undetermined int |
|  | ICD-10-AM | Y11 | Poisoning by and exposure to antiepileptic, sedative-hypnotic, antiparkinsonism and psychotropic dru |
|  | ICD-10-AM | Y12 | Poisoning by and exposure to narcotics and psychodysleptics [hallucinogens], not elsewhere classifie |
|  | ICD-10-AM | Y13 | Poisoning by and exposure to other drugs acting on the autonomic nervous system, undetermined intent |
|  | ICD-10-AM | Y14 | Poisoning by and exposure to other and unspecified drugs, medicaments and biological substances, und |
|  | ICD-10-AM | Y15 | Poisoning by and exposure to alcohol, undetermined intent |
|  | ICD-10-AM | Y16 | Poisoning by and exposure to organic solvents and halogenated hydrocarbons and their vapours, undete |
|  | ICD-10-AM | Y18 | Poisoning by and exposure to pesticides, undetermined intent |
|  | ICD-10-AM | Y19 | Poisoning by and exposure to other and unspecified chemicals and noxious substances, undetermined in |
|  | SNOMED CT | Descendants of 1149222004\|Overdose\| that do not include 'Intentional' or 'Accidental' | 5-aminosalicylic acid overdose |
|  | SNOMED CT | 11196001 | Opiate agonist poisoning |
| flag_APDC_unk_pois  flag_ED_unk_pois | ICD-10-AM | T40 | Poisoning by narcotics and psychodysleptics [hallucinogens] |
|  | ICD-10-AM | T41 | Poisoning by anaesthetics and therapeutic gases |
|  | ICD-10-AM | T42 | Poisoning by antiepileptic, sedative-hypnotic and antiparkinsonism drugs |
|  | ICD-10-AM | T43 | Poisoning by psychotropic drugs, not elsewhere classified |
|  | ICD-10-AM | T51 | Toxic effect of alcohol |
|  | ICD-9 | 305 | Nondependent alcohol abuse |
|  | ICD-9 | 977 | Poisoning by other and unspecified drugs and medicinal substances |
|  | ICD-9 | 969 | Poisoning by psychotropic agents |
|  | ICD-9 | 965 | Poisoning by analgesics antipyretics and antirheumatics |
|  | SNOMED CT | 7895008\|Drug toxicity\| and its decendants that do not contain 'Accidental' or 'Intentional' | 4-aminopyridine poisoning |

### Table S5 Criminal charges derivation

| Conviction type | ANZSOC code |
| --- | --- |
| Violent | 0111 Murder |
|  | 0121 Attempted murder |
|  | 0131 Manslaughter |
|  | 0132 Driving causing death |
|  | 0211 Serious assault resulting in injury |
|  | 0212 Serious assault not resulting in injury |
|  | 0213 Common assault |
|  | 0291 Stalking |
|  | 0299 Other acts intended to cause injury, nec |
|  | 0311 Aggravated sexual assault |
|  | 0312 Non-aggravated sexual assault |
|  | 0511 Abduction and kidnapping |
|  | 0521 Deprivation of liberty/false imprisonment |
|  | 0532 Threatening behaviour |
|  | 0611 Aggravated robbery |
|  | 0612 Non-aggravated robbery |
| NonViolent | 0711 Unlawful entry with intent/burglary, break and enter |
|  | 0811 Theft of a motor vehicle |
|  | 0812 Illegal use of a motor vehicle |
|  | 0813 Theft of motor vehicle parts or contents |
|  | 0821 Theft from a person (excluding by force) |
|  | 0822 Theft of intellectual property |
|  | 0823 Theft from retail premises |
|  | 0829 Theft (except motor vehicles), nec |
|  | 0841 Illegal use of property (except motor vehicles) |
|  | 0911 Obtain benefit by deception |
|  | 0921 Counterfeiting of currency |
|  | 0922 Forgery of documents |
|  | 0923 Possess equipment to make false/illegal instrument |
|  | 1011 Import illicit drugs |
|  | 1012 Export illicit drugs |
|  | 1021 Deal or traffic in illicit drugs - commercial quantity |
|  | 1022 Deal or traffic in illicit drugs - non-commercial quantity |
|  | 1031 Manufacture illicit drugs |
|  | 1032 Cultivate illicit drugs |
|  | 1041 Possess illicit drugs |
|  | 1042 Use illicit drugs |
|  | 1099 Other illicit drug offences, nec |

### Table S6: Dimension reduction – results from decision tree

| Variable Name | Number  of Splitting  Rules | Importance | Validation Importance | Ratio of Validation  to Training Importance | Decision to use in the next stage |
| --- | --- | --- | --- | --- | --- |
| flag_APDC_int_od_12 | 1 | 1 | 1 | 1 | Keep |
| elapsed_time | 93 | 0.709 | 0.2554 | 0.3603 | Keep |
| NumED_6mon | 18 | 0.6478 | 0.6331 | 0.9773 | Keep |
| flag_APDC_bpd_6 | 2 | 0.6041 | 0.6905 | 1.143 | Keep |
| IRSD_Decile_State | 61 | 0.5787 | 0.1147 | 0.1982 | Keep |
| age | 54 | 0.4927 | 0.089 | 0.1806 | Keep |
| NumED_12mon | 40 | 0.4539 | 0.3343 | 0.7365 | Drop |
| Num_NonViolentCharges_12mon | 18 | 0.3702 | 0.0885 | 0.239 | Keep |
| NumED_3mon | 17 | 0.3556 | 0.1997 | 0.5617 | Drop |
| num_prescriber_12mon | 19 | 0.3106 | 0.1494 | 0.4811 | Keep |
| flag_ed_unk_od_3 | 3 | 0.282 | 0.1141 | 0.4046 | Keep |
| flag_APDC_si_inj_12 | 5 | 0.2804 | 0.2254 | 0.8037 | Keep |
| flag_APDC_bpd_12 | 3 | 0.2708 | 0.2501 | 0.9238 | Drop |
| flag_APDC_depanx_12 | 10 | 0.2702 | 0.2035 | 0.7529 | Keep |
| flag_Procedure_3mon | 6 | 0.2463 | 0.1004 | 0.4077 | Keep |
| flag_APDC_dep_oth_12 | 7 | 0.2411 | 0.1396 | 0.5788 | Keep |
| flag_APDC_unk_od_12 | 5 | 0.2399 | 0.0372 | 0.1549 | Keep |
| flag_MHAmb_persondisorder_3 | 2 | 0.2374 | 0.1731 | 0.7291 | Keep |
| flag_APDC_ho_3 | 6 | 0.2357 | 0.1437 | 0.6098 | Keep |
| num_OATS_12mon | 14 | 0.2325 | 0.1046 | 0.4498 | Keep |
| flag_APDC_dep_alc_12 | 4 | 0.2265 | 0.0578 | 0.2553 | Keep |
| OAT_90 | 5 | 0.2259 | 0 | 0 | Keep |
| flag_APDC_persondisorder_6 | 3 | 0.2195 | 0 | 0 | Drop |
| flag_MHAmb_dep_opi_12 | 7 | 0.2172 | 0.0089 | 0.0409 | Keep |
| num_OATS_3mon | 6 | 0.2147 | 0.0541 | 0.2521 | Drop |
| flag_APDC_home_12 | 4 | 0.2044 | 0.0886 | 0.4332 | Keep |
| Num_NonViolentConvictions_12mon | 7 | 0.2007 | 0.0287 | 0.1432 | Keep |
| OAT_30 | 8 | 0.1948 | 0.0676 | 0.3471 | Drop |
| flag_APDC_si_inj_3 | 2 | 0.1941 | 0.1984 | 1.0219 | Drop |
| flag_APDC_dep_opi_6 | 6 | 0.194 | 0.0319 | 0.1645 | Keep |
| flag_APDC_dep_opi_12 | 7 | 0.1928 | 0.1146 | 0.5942 | Drop |
| flag_APDC_dep_oth_6 | 5 | 0.1881 | 0.0678 | 0.3602 | Drop |
| Sex | 10 | 0.185 | 0.0505 | 0.2732 | Keep |
| num_prescriber_6mon | 6 | 0.1813 | 0 | 0 | Drop |
| num_OATS_6mon | 6 | 0.1778 | 0.0513 | 0.2884 | Drop |
| flag_APDC_dep_oth_3 | 5 | 0.1757 | 0 | 0 | Drop |
| flag_APDC_dep_sed_6 | 2 | 0.1722 | 0 | 0 | Keep |
| flag_MHAmb_depanx_3 | 5 | 0.1716 | 0.0459 | 0.2675 | Keep |
| flag_APDC_idea_3 | 3 | 0.1689 | 0.0433 | 0.2561 | Keep |
| flag_APDC_dep_alc_3 | 5 | 0.1687 | 0 | 0 | Drop |
| Num_ViolentCharges_6mon | 6 | 0.1624 | 0.0606 | 0.3731 | Keep |
| flag_APDC_ho_12 | 5 | 0.1616 | 0.1011 | 0.6257 | Drop |
| flag_APDC_persondisorder_12 | 4 | 0.1613 | 0.0878 | 0.544 | Keep |
| flag_incar_12M | 5 | 0.1592 | 0.0627 | 0.394 | Keep |
| flag_ed_unk_pois_12 | 2 | 0.1587 | 0 | 0 | Keep |
| Num_NonViolentCharges_6mon | 5 | 0.1584 | 0.0875 | 0.5525 | Drop |
| flag_APDC_home_3 | 5 | 0.1543 | 0.0592 | 0.384 | Drop |
| flag_MHAmb_persondisorder_12 | 4 | 0.1533 | 0.0372 | 0.2429 | Drop |
| flag_APDC_unk_pois_12 | 3 | 0.1525 | 0.0367 | 0.2405 | Keep |
| flag_MHAmb_dep_opi_6 | 3 | 0.1483 | 0 | 0 | Keep |
| Num_NonViolentCharges_3mon | 5 | 0.1476 | 0 | 0 | Drop |
| flag_incar_3M | 4 | 0.1475 | 0.0389 | 0.2636 | Drop |
| OAT_60 | 4 | 0.1468 | 0.0409 | 0.2786 | Drop |
| flag_APDC_dep_sed_3 | 5 | 0.1412 | 0 | 0 | Drop |
| flag_APDC_int_od_6 | 3 | 0.136 | 0.0544 | 0.3999 | Drop |
| Num_NonViolentConvictions_6mon | 4 | 0.1355 | 0 | 0 | Drop |
| flag_APDC_acc_od_12 | 5 | 0.1334 | 0.0513 | 0.3844 | Keep |
| flag_APDC_idea_6 | 2 | 0.1322 | 0.0633 | 0.4789 | Drop |
| flag_Procedure_12mon | 2 | 0.1301 | 0 | 0 | Drop |
| flag_APDC_idea_12 | 2 | 0.1298 | 0.1496 | 1.1531 | Drop |
| flag_APDC_dep_sed_12 | 2 | 0.1275 | 0 | 0 | Drop |
| flag_APDC_dep_alc_6 | 3 | 0.1217 | 0.0622 | 0.5106 | Drop |
| flag_MHAmb_persondisorder_6 | 1 | 0.1208 | 0 | 0 | Drop |
| flag_ed_psyc_3 | 3 | 0.1206 | 0 | 0 | Keep |
| flag_MHAmb_psyc_12 | 3 | 0.1206 | 0.0449 | 0.3724 | Keep |
| flag_change_DosingPointSector | 2 | 0.1198 | 0.0865 | 0.7221 | Keep |
| flag_APDC_acc_od_6 | 3 | 0.1149 | 0.0298 | 0.2594 | Drop |
| flag_APDC_bpd_3 | 2 | 0.1149 | 0 | 0 | Drop |
| aria | 4 | 0.1129 | 0.0156 | 0.1383 | Keep |
| flag_APDC_unk_od_3 | 1 | 0.1128 | 0 | 0 | Drop |
| flag_Procedure_6mon | 2 | 0.1111 | 0 | 0 | Drop |
| indig_fg | 3 | 0.111 | 0 | 0 | Keep |
| flag_APDC_irrit_6 | 1 | 0.1102 | 0 | 0 | Keep |
| flag_ed_idea_12 | 2 | 0.1053 | 0.0663 | 0.6293 | Keep |
| flag_APDC_dep_stim_3 | 2 | 0.1021 | 0.0477 | 0.4676 | Keep |
| flag_MHAmb_dep_alc_6 | 2 | 0.1003 | 0 | 0 | Keep |
| flag_APDC_irrit_3 | 1 | 0.0978 | 0 | 0 | Drop |
| flag_ed_depanx_3 | 2 | 0.0978 | 0 | 0 | Keep |
| flag_incar_6M | 4 | 0.0976 | 0 | 0 | Drop |
| flag_MHAmb_psyc_6 | 1 | 0.0973 | 0.072 | 0.7398 | Drop |
| flag_ed_dep_opi_3 | 1 | 0.0952 | 0 | 0 | Keep |
| flag_ed_depanx_6 | 2 | 0.0935 | 0 | 0 | Drop |
| Num_ViolentCharges_12mon | 2 | 0.093 | 0.0987 | 1.0618 | Drop |
| flag_MHAmb_dep_alc_12 | 3 | 0.0925 | 0.0336 | 0.3634 | Drop |
| flag_APDC_psyc_12 | 3 | 0.0921 | 0 | 0 | Keep |
| flag_APDC_int_pois_12 | 1 | 0.0911 | 0.07 | 0.7684 | Keep |
| flag_ed_dep_alc_12 | 1 | 0.0903 | 0 | 0 | Keep |
| flag_APDC_dep_opi_3 | 1 | 0.0897 | 0 | 0 | Drop |
| flag_ed_dep_oth_12 | 1 | 0.0897 | 0 | 0 | Keep |
| flag_MHAmb_idea_3 | 2 | 0.0896 | 0.1152 | 1.2858 | Keep |
| flag_APDC_unk_inj_12 | 1 | 0.0888 | 0 | 0 | Keep |
| flag_APDC_dep_stim_6 | 1 | 0.0875 | 0 | 0 | Drop |
| flag_MHAmb_depanx_12 | 1 | 0.0866 | 0 | 0 | Drop |
| flag_APDC_dep_stim_12 | 2 | 0.0862 | 0.0858 | 0.9956 | Drop |
| flag_APDC_int_od_3 | 1 | 0.0858 | 0 | 0 | Drop |
| flag_ed_depanx_12 | 3 | 0.0855 | 0.0523 | 0.6113 | Drop |
| flag_APDC_depanx_3 | 2 | 0.0848 | 0 | 0 | Drop |
| flag_APDC_persondisorder_3 | 2 | 0.0837 | 0 | 0 | Drop |
| flag_APDC_depanx_6 | 2 | 0.0809 | 0.0384 | 0.4744 | Drop |
| num_prescriber_3mon | 1 | 0.0796 | 0 | 0 | Drop |
| flag_APDC_dep_coc_6 | 1 | 0.0792 | 0 | 0 | Keep |
| flag_APDC_home_6 | 1 | 0.078 | 0 | 0 | Drop |
| flag_ed_dep_opi_6 | 1 | 0.0772 | 0 | 0 | Drop |
| flag_ed_psyc_12 | 3 | 0.0733 | 0 | 0 | Drop |
| flag_MHAmb_bpd_12 | 2 | 0.0729 | 0.0235 | 0.3228 | Keep |
| flag_ed_idea_3 | 2 | 0.07 | 0 | 0 | Drop |
| flag_APDC_ho_6 | 1 | 0.0684 | 0 | 0 | Drop |
| Num_ViolentConvictions_6mon | 1 | 0.0663 | 0 | 0 | Keep |
| flag_APDC_psyc_3 | 1 | 0.0641 | 0 | 0 | Drop |
| Num_ViolentConvictions_3mon | 1 | 0.063 | 0 | 0 | Drop |
| flag_MHAmb_ptsd_6 | 1 | 0.0622 | 0 | 0 | Drop |
| flag_ed_cpain_12 | 1 | 0.0615 | 0 | 0 | Keep |
| flag_APDC_int_pois_6 | 1 | 0.0591 | 0 | 0 | Drop |
| flag_ed_dep_stim_3 | 1 | 0.0579 | 0 | 0 | Keep |
| flag_APDC_unk_pois_6 | 1 | 0.0578 | 0.1028 | 1.7782 | Drop |
| flag_APDC_irrit_12 | 1 | 0.0547 | 0 | 0 | Drop |
| flag_MHAmb_dep_oth_6 | 1 | 0.0532 | 0 | 0 | Keep |
| flag_MHAmb_depanx_6 | 1 | 0.0521 | 0 | 0 | Drop |
| flag_ed_psyc_6 | 1 | 0.0512 | 0.0315 | 0.615 | Drop |
| flag_APDC_ptsd_12 | 1 | 0.0488 | 0 | 0 | Drop |
| Num_NonViolentConvictions_3mon | 2 | 0.0448 | 0.0625 | 1.3951 | Drop |
| Num_ViolentConvictions_12mon | 1 | 0.0402 | 0.0296 | 0.7378 | Drop |
| flag_ed_dep_oth_6 | 1 | 0.034 | 0.0559 | 1.6412 | Drop |
| flag_APDC_dep_coc_12 | 1 | 0.0321 | 0 | 0 | Drop |
| flag_MHAmb_dep_oth_12 | 1 | 0.03 | 0 | 0 | Drop |

### Table S7: Machine learning algorithm properties

| Algorithm | property | Value |
| --- | --- | --- |
| Decision Tree | Splitting criterion | Chi-square probability |
| (Variable Reduction) | Significance level | 0.2 |
|  | Maximum branch | 2 |
|  | Maximum depth | 10 |
|  | Leaf size | 5 |
|  | Number of surrogate rules | 5 |
|  | Method | Largest tree (no pruning) |
|  | Bonferonni adjustment | Before the split was chosen |
|  | Depth adjustment | Yes |
| Gradient Boosting | Shrinkage | 0.01 |
|  | Train proportion | 80 |
|  | Maximum depth | 5 |
|  | Reuse variable | 3 |
|  | Leaf fraction | 0.001 |
|  | N iterations | 760 |
| Neural Network | Type | Multilayer perceptron |
|  | Direct connections | Yes |
|  | Hidden units | 5 |
| Logistic Regression | Method | Stepwise |
|  | Entry | 0.15 |
|  | Stay | 0.05 |
|  | Polynomials | Power of 2 |
|  | Two factor interactions | Yes |
| Ensemble | Method | Average |

## Supplementary 2 - Results

### Table S8: Model goodness of fit statistics

| **Model Description** | **Test: Gini**  **Coefficient** | **Train: Roc**  **Index** | **Train: Gini**  **Coefficient** | **Valid: Roc**  **Index** | **Valid: Gini**  **Coefficient** | **Test: Roc**  **Index** |
| --- | --- | --- | --- | --- | --- | --- |
| Gradient Boosting | 0.651 | 0.829 | 0.658 | 0.823 | 0.646 | 0.825 |
| Ensemble | 0.642 | 0.825 | 0.649 | 0.821 | 0.643 | 0.821 |
| Logistic regression (stepwise) | 0.610 | 0.811 | 0.621 | 0.809 | 0.618 | 0.805 |
| Neural Network | 0.604 | 0.809 | 0.618 | 0.804 | 0.607 | 0.802 |

The gradient boosting algorithm had the highest Gini coefficient based on the test (holdout) dataset, this model differentiates best between those that will self-harm/suicide from those that will not.

### Table S9: Cohort characteristics at first prediction window

| **Characteristics per person first prediction window in training data** | **N (%)** |
| --- | --- |
|  |  |
| Number of participants | 45828 |
| Age(yrs) | 34 IQR (28-42) |
| Females | 14,899 (32.5%) |
| Identified as Aboriginal or Torres Strait Islander | 9,546 (20.8%) |
| Number emergency visits in the last 12 months | 0 IQR (0-1) |
| Hospital admission for intentional overdose in the last 12 months | 605 (1.3%) |
| Hospital admission for unknown intent poisoning in the last 12 months | 1,113 (2.4%) |
| Hospital admission for borderline personality disorder in the last 12 months | 560 (1.2%) |
| Hospital admission for depression or anxiety in the last 12 months | 2,077 (4.5%) |
| Number non-violent charges in the last 12 months | 0 IQR (0-1) |
| Released from incarceration in the last 12 months | 6,627 (14.5%) |

### Table S10: Gradient boosting important features

| **Variable Name** | **No. of Splitting Rules** | **Importance** | **Ratio of Validation to Training** | **Interaction Importance** |
| --- | --- | --- | --- | --- |
| Intentional overdose (APDC-Ind-12M) | 214 | 1.000 | 1.000 | 0.053 |
| Number of ED presentations (EDDC-Num-6M) | 879 | 0.803 | 1.108 | 0.090 |
| Unknown intent poisoning (APDC-Ind-12M) | 243 | 0.519 | 1.032 | 0.019 |
| Depression/Anxiety (APDC-Ind-3M) | 354 | 0.468 | 1.001 | 0.032 |
| Outpatient personality disorder (MHAmb-Ind-3M) | 205 | 0.429 | 0.766 | 0.030 |
| Socio-economic disadvantage index | 1058 | 0.421 | 0.178 | 0.021 |
| Time since OAT initiation in days | 763 | 0.341 | 0.594 | 0.014 |
| Any hospital procedure (APDC-Ind-3M) | 218 | 0.271 | 0.587 | 0.012 |
| Other substance dependence (APDC-Ind-12M) | 183 | 0.234 | 1.003 | 0.003 |
| Outpatient opioid dependence (MHAmb-Ind-12M) | 220 | 0.222 | 0.632 | 0.004 |
| Alcohol dependence (APDC-Ind-12M) | 171 | 0.208 | 0.998 | 0.003 |
| Borderline personality disorder (APDC-Ind-6M) | 12 | 0.200 | 1.089 | 0.001 |
| Number OAT prescribers 12M | 276 | 0.193 | 0.318 | 0.002 |
| Age | 304 | 0.188 | 0.517 | 0.001 |
| Psychosis (APDC-Ind-12M) | 148 | 0.185 | 1.158 | 0.002 |
| Homelessness (APDC-Ind-12M) | 123 | 0.183 | 0.981 | 0.002 |
| Sedative dependence (APDC-Ind-6M) | 103 | 0.158 | 0.990 | 0.002 |
| Outpatient psychosis (MHAmb-Ind-12M) | 130 | 0.148 | 0.761 | 0.001 |
| Opioid dependence (APDC-Ind-6M) | 88 | 0.130 | 0.893 | 0.001 |
| Sex | 85 | 0.128 | 0.250 | 0.003 |
| Outpatient depression/anxiety (MHAmb-Ind-3M) | 94 | 0.120 | 0.452 | 0.001 |
| OAT days last 90 days | 137 | 0.108 | 0.528 | 0.000 |
| Number of days on OAT (ERRCD-Num-12M) | 113 | 0.108 | 0.314 | 0.000 |
| Unknown intent overdose (APDC-Ind-12M) | 20 | 0.107 | 0.283 | 0.000 |
| Number Non-violent convictions 12M | 97 | 0.091 | 0.528 | 0.000 |
| Release from incarceration (ROD-Ind-12M) | 53 | 0.077 | 0.890 | 0.000 |
| Personality disorder (APDC-Ind-6M) | 7 | 0.052 | 0.346 | 0.000 |
| Self-inflicted injury (APDC-Ind-12M) | 1 | 0.047 | 1.144 | 0.000 |
| Number violent charges 6M | 13 | 0.033 | 0.323 | 0.000 |
| Stimulant dependence (APDC-Ind-3M) | 5 | 0.023 | 1.258 | 0.000 |

Note: (*DATA source-variable type-time period*). The importance of a variable is the contribution it makes to the success of the model. A high ratio of validation to training importance for a variable suggests that the variable is important not only in the training data but also in the validation data, indicating good generalization.

### Table S11 Model net benefit, sensitivity and specificity per threshold probability

| **Threshold probability (%)** | **Monthly average identified high risk by model** | **Sensitivity** | **Specificity** | **Net Benefit - Gradient boosting** | **Net Benefit - treatment for all** | **Net Benefit - Marker 1** | **Net Benefit - Marker 2** | **Net Benefit - Markers 1 & 2** | **Captured response - Gradient boosting** |
| --- | --- | --- | --- | --- | --- | --- | --- | --- | --- |
| 0 | 27940 | 1.00 | 0.00 | 0.00181 | 0.00181 | 0.00093 | 0.00038 | 0.00100 | 1.000 |
| 0.03 | 27940 | 1.00 | 0.50 | 0.00151 | 0.00151 | 0.00089 | 0.00038 | 0.00096 | 1.000 |
| 0.06 | 27940 | 1.00 | 0.50 | 0.00121 | 0.00121 | 0.00086 | 0.00037 | 0.00093 | 1.000 |
| 0.09 | 9252 | 0.82 | 0.75 | 0.00118 | 0.00091 | 0.00083 | 0.00037 | 0.00090 | 0.817 |
| 0.12 | 7540 | 0.77 | 0.79 | 0.00107 | 0.00061 | 0.00080 | 0.00037 | 0.00086 | 0.772 |
| **0.15** | **5167** | **0.69** | **0.84** | **0.00098** | **0.00031** | **0.00076** | **0.00036** | **0.00083** | **0.695** |
| 0.18 | 4724 | 0.67 | 0.86 | 0.00091 | 0.00001 | 0.00073 | 0.00036 | 0.00079 | 0.670 |
| 0.21 | 4402 | 0.66 | 0.86 | 0.00086 | -0.00029 | 0.00070 | 0.00035 | 0.00076 | 0.655 |
| 0.24 | 3570 | 0.61 | 0.89 | 0.00080 | -0.00059 | 0.00066 | 0.00035 | 0.00072 | 0.614 |
| 0.27 | 2988 | 0.59 | 0.90 | 0.00077 | -0.00090 | 0.00063 | 0.00035 | 0.00069 | 0.586 |
| 0.3 | 2632 | 0.56 | 0.91 | 0.00074 | -0.00120 | 0.00060 | 0.00034 | 0.00065 | 0.564 |
| 0.33 | 2327 | 0.54 | 0.92 | 0.00071 | -0.00150 | 0.00057 | 0.00034 | 0.00062 | 0.543 |
| 0.36 | 2099 | 0.52 | 0.93 | 0.00068 | -0.00180 | 0.00053 | 0.00034 | 0.00058 | 0.523 |
| 0.39 | 1893 | 0.51 | 0.94 | 0.00066 | -0.00210 | 0.00050 | 0.00033 | 0.00055 | 0.512 |
| 0.42 | 1724 | 0.49 | 0.94 | 0.00064 | -0.00240 | 0.00047 | 0.00033 | 0.00052 | 0.493 |
| 0.45 | 1594 | 0.48 | 0.95 | 0.00062 | -0.00271 | 0.00043 | 0.00032 | 0.00048 | 0.484 |
| 0.48 | 1485 | 0.46 | 0.95 | 0.00059 | -0.00301 | 0.00040 | 0.00032 | 0.00045 | 0.464 |
| 0.51 | 1398 | 0.45 | 0.95 | 0.00057 | -0.00331 | 0.00037 | 0.00032 | 0.00041 | 0.454 |
| 0.54 | 1315 | 0.45 | 0.96 | 0.00056 | -0.00361 | 0.00033 | 0.00031 | 0.00038 | 0.451 |
| 0.57 | 1246 | 0.45 | 0.96 | 0.00055 | -0.00392 | 0.00030 | 0.00031 | 0.00034 | 0.446 |
| 0.6 | 1185 | 0.44 | 0.96 | 0.00054 | -0.00422 | 0.00027 | 0.00030 | 0.00031 | 0.436 |
| 0.63 | 1122 | 0.43 | 0.96 | 0.00052 | -0.00452 | 0.00024 | 0.00030 | 0.00027 | 0.426 |
| 0.66 | 1071 | 0.41 | 0.96 | 0.00049 | -0.00482 | 0.00020 | 0.00030 | 0.00024 | 0.408 |
| 0.69 | 1024 | 0.40 | 0.97 | 0.00048 | -0.00513 | 0.00017 | 0.00029 | 0.00020 | 0.404 |
| 0.72 | 980 | 0.40 | 0.97 | 0.00047 | -0.00543 | 0.00014 | 0.00029 | 0.00017 | 0.398 |
| 0.75 | 944 | 0.39 | 0.97 | 0.00046 | -0.00574 | 0.00010 | 0.00029 | 0.00013 | 0.391 |
| 0.78 | 906 | 0.39 | 0.97 | 0.00045 | -0.00604 | 0.00007 | 0.00028 | 0.00010 | 0.388 |
| 0.81 | 870 | 0.38 | 0.97 | 0.00045 | -0.00634 | 0.00004 | 0.00028 | 0.00006 | 0.384 |
| 0.84 | 838 | 0.37 | 0.97 | 0.00043 | -0.00665 | 0.00000 | 0.00027 | 0.00003 | 0.373 |
| 0.87 | 811 | 0.36 | 0.97 | 0.00041 | -0.00695 | -0.00003 | 0.00027 | -0.00001 | 0.365 |
| 0.9 | 782 | 0.36 | 0.97 | 0.00041 | -0.00726 | -0.00006 | 0.00027 | -0.00004 | 0.361 |
| 0.93 | 755 | 0.35 | 0.97 | 0.00039 | -0.00756 | -0.00010 | 0.00026 | -0.00008 | 0.355 |
| 0.96 | 729 | 0.35 | 0.98 | 0.00039 | -0.00787 | -0.00013 | 0.00026 | -0.00011 | 0.350 |
| 0.99 | 704 | 0.35 | 0.98 | 0.00038 | -0.00817 | -0.00016 | 0.00025 | -0.00015 | 0.347 |

At a threshold probability of 0.15, i.e. a probability of self-harm/suicide >= 0.15, the model identified 5167 at high risk, with sensitivity of 69% and specificity of 84%. The net benefit had a value of 0.00098 for the predictive model compared to 0.00031 for the screen for all approach.

Figure S1: Prediction model process of self-harm/suicide within any 30-day window


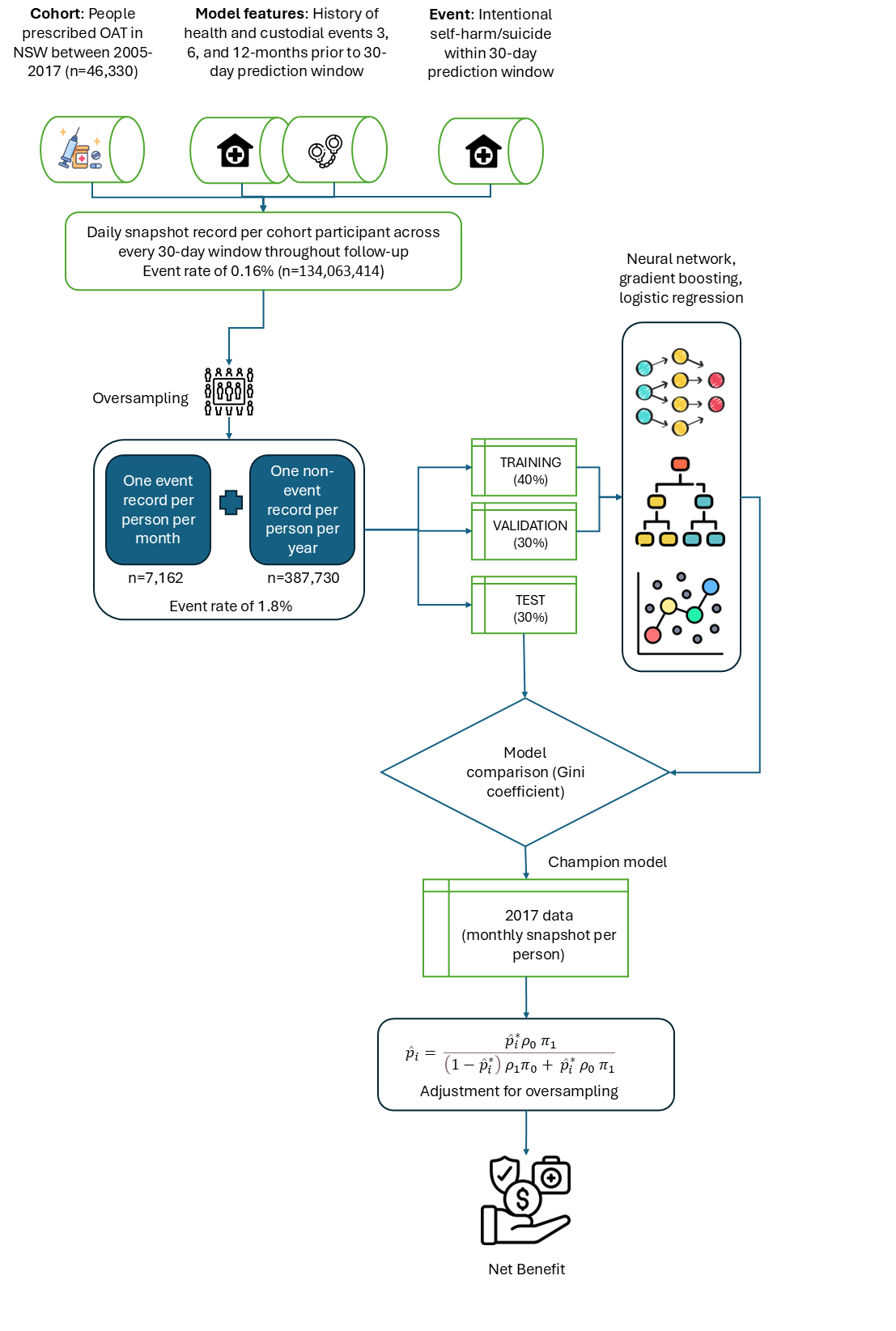


Figure S2 Sensitivity versus 1-specificity (ROC) chart


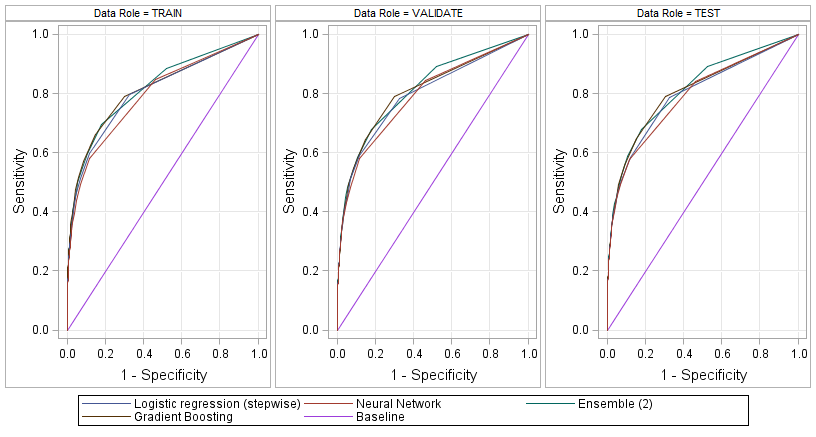
Note: Sensitivity, [# of true positives/(# of true positives + # of false negatives)], is the ability of a model to correctly classify an individual as being hospitalised for self-harm within a 30-day window. Specificity the ability of a model to correctly classify an individual as NOT being hospitalised for self-harm or death by suicide within a 30-day window, [# of true negatives/(# of true negatives + # of false positives)].

### Figure S3 Decision tree output, predicting the gradient boosting outcome using a sample (n=10000)


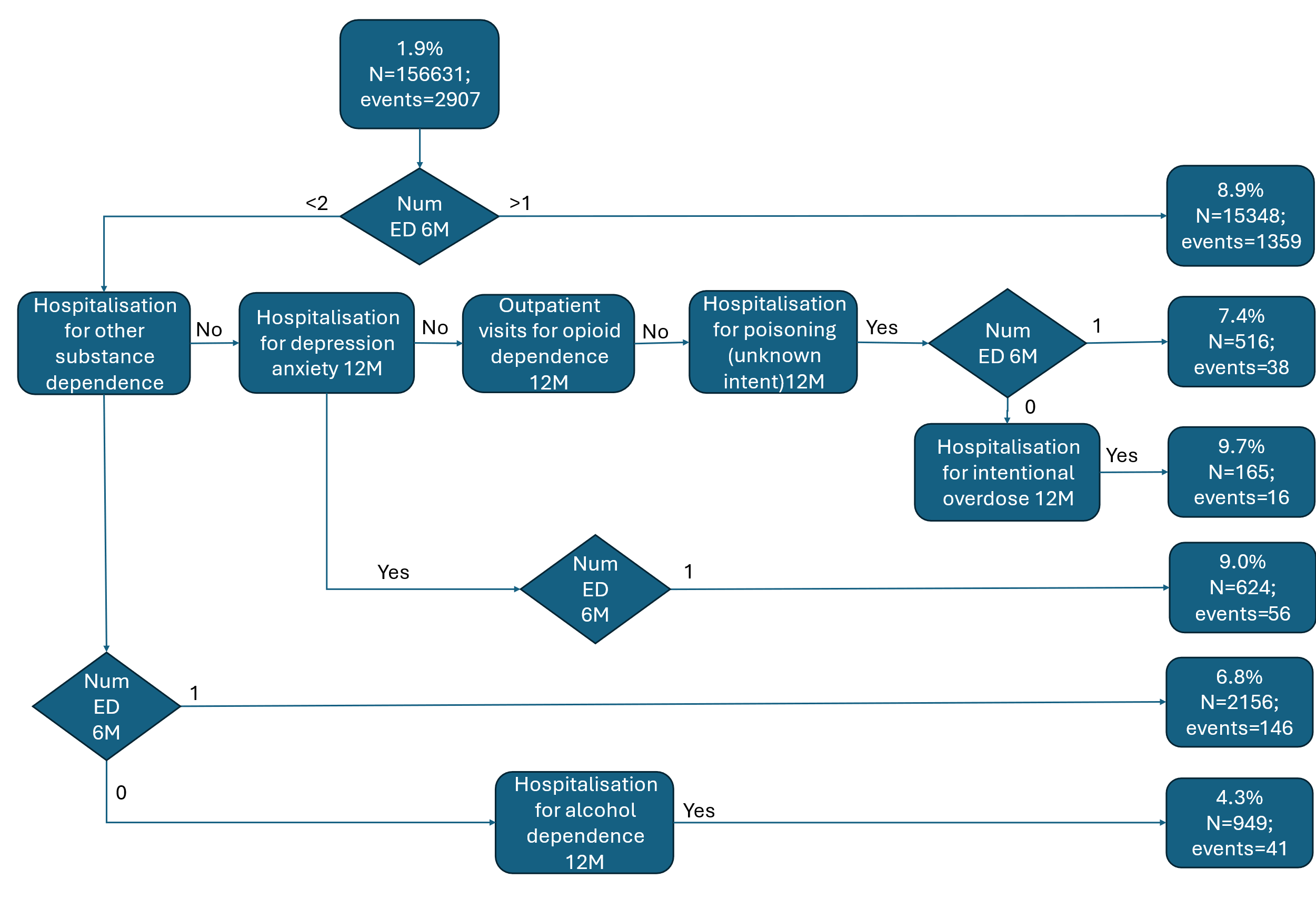


### Figure S4 Individual B - SHAP waterfall


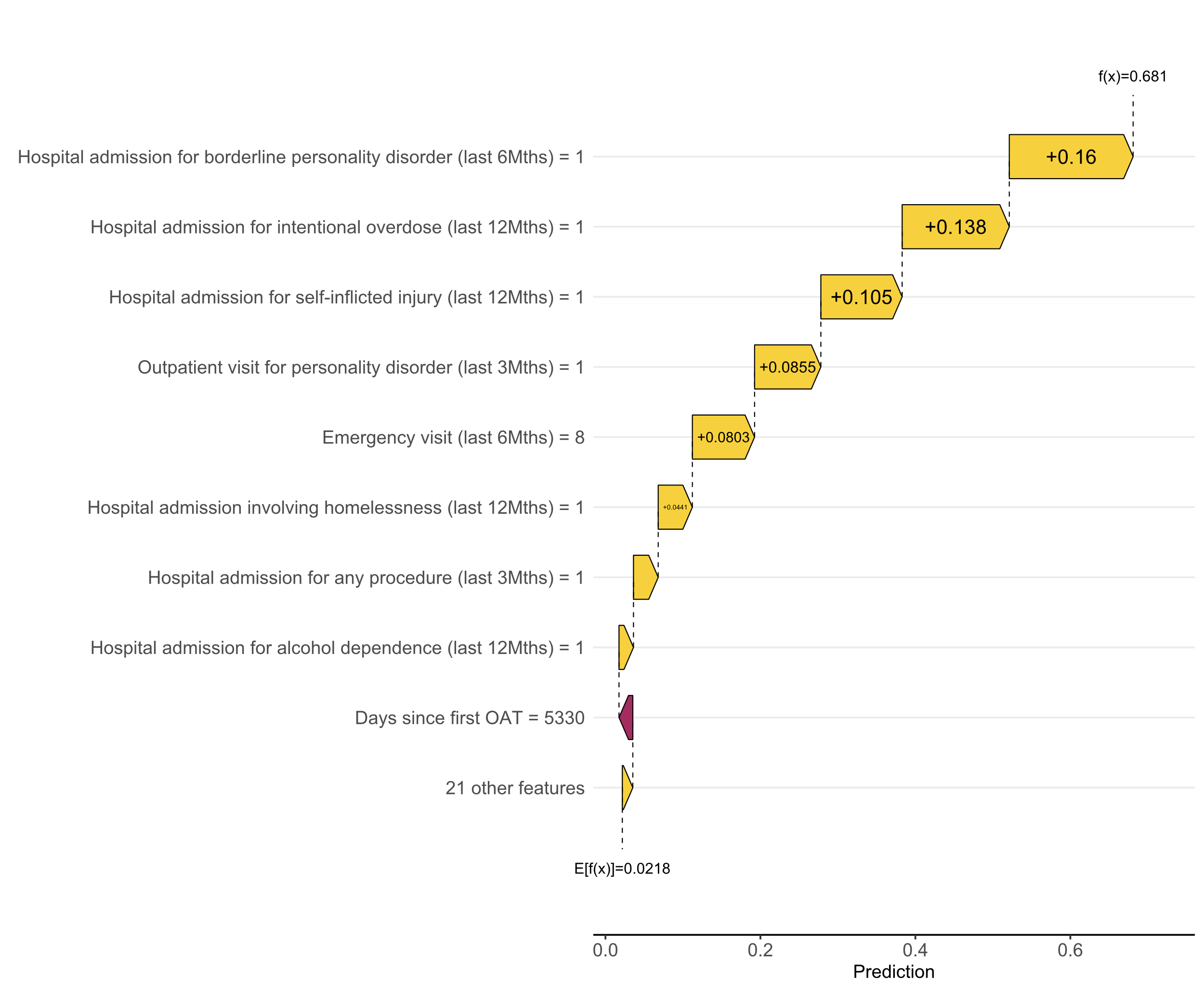


This individual had an expected probability to self-harm/suicide of 0.681 much higher than the baseline risk of 0.0218. The top factors that contributed to this high risk were a hospitalisation with a diagnosis of borderline personality disorder in the last 6 months, a hospitalisation for an intentional overdose in the last 12 months as well as self-inflicted injury.

### Figure S5 Individual C - SHAP waterfall


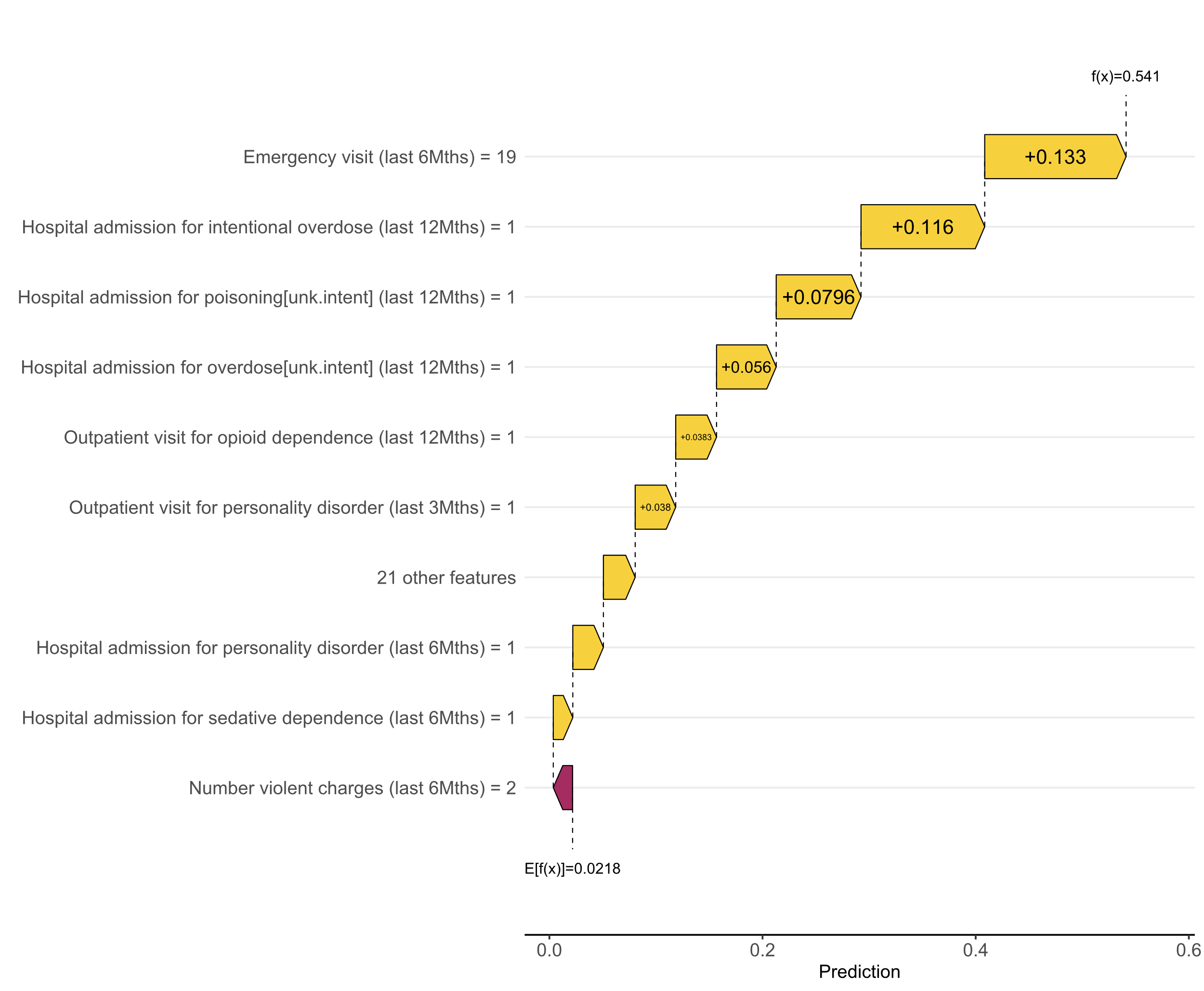
 This individual had an expected probability to self-harm/suicide of 0.541, the top factors that contributed to this high risk were 19 ED visits in the last 6 months, a hospitalisation for an intentional overdose in the last 12 months as well as poisoning (unknown intent).

### Figure S6 Individual D - SHAP waterfall


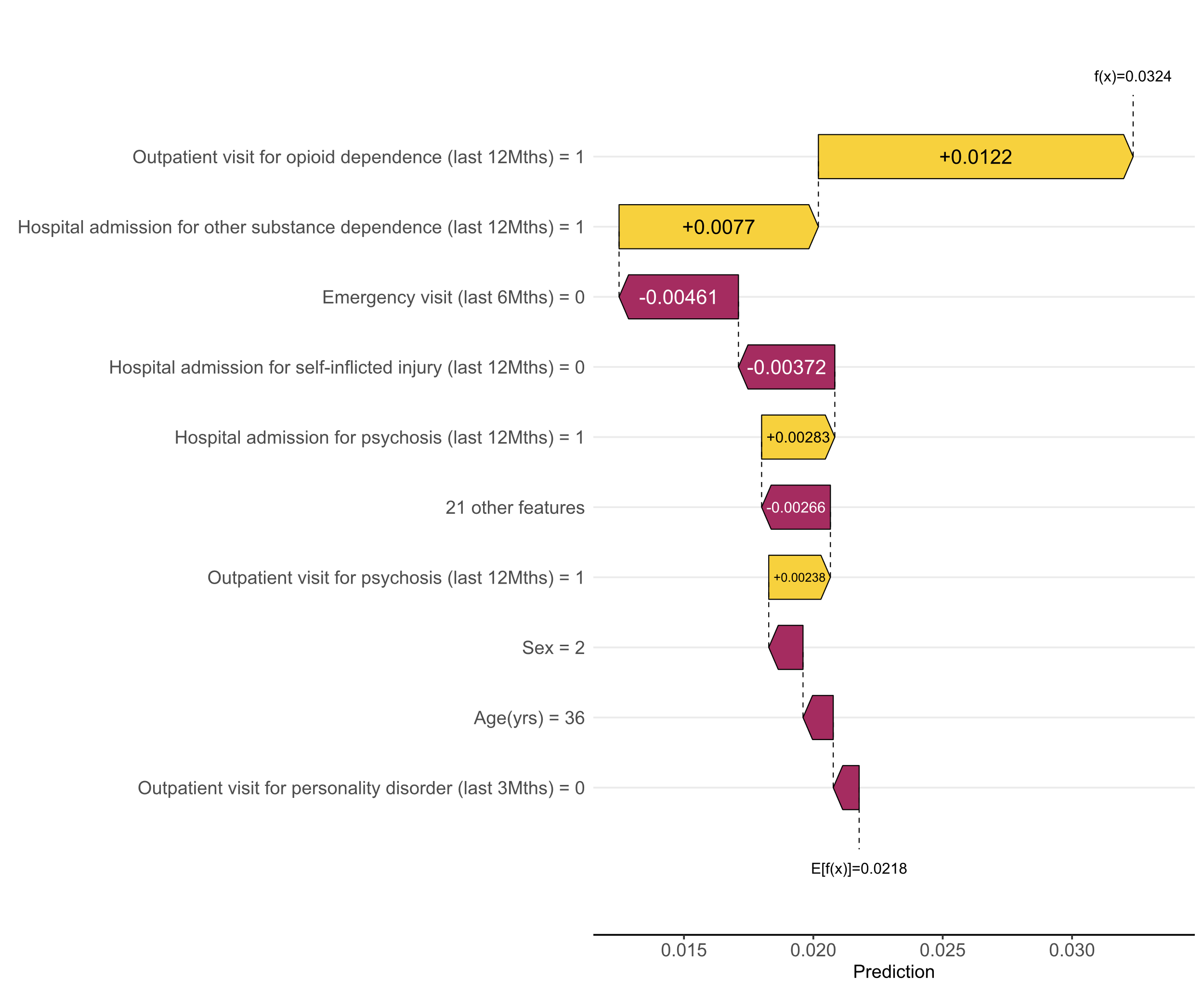


This individual had an expected probability to self-harm/suicide of 0.0324, the top factors that contributed to a higher risk than baseline (0.0218) were an outpatient visit for opioid dependence, recent hospitalisation for substance dependence and psychosis. Having no ED visits in the last 6 months reduced their propensity score.

### References

1. Schröer C, Kruse F, Gómez JM. A Systematic Literature Review on Applying CRISP-DM Process Model. Procedia Comput Sci. 2021;181:526-34.

2. Erickson BJ, Kitamura F. Magician's Corner: 9. Performance Metrics for Machine Learning Models. Radiol Artif Intell. 2021;3(3):e200126.

3. Vickers AJ, Van Calster B, Steyerberg EW. Net benefit approaches to the evaluation of prediction models, molecular markers, and diagnostic tests. BMJ. 2016;352:i6.

4. Shapley LS. 17. A Value for n-Person Games. In: Harold William K, Albert William T, editors. Contributions to the Theory of Games, Volume II. Princeton: Princeton University Press; 1953. p. 307-18.

5. Lundberg SM, Lee S-I. A unified approach to interpreting model predictions. Proceedings of the 31st International Conference on Neural Information Processing Systems; Long Beach, California, USA: Curran Associates Inc.; 2017. p. 4768–77.
